# Western Diet and fecal microbiota transplantation alter phenotypic, liver fatty acids, and gut metagenomics and metabolomics in Mtarc2 knockout mice

**DOI:** 10.1186/s12263-025-00772-x

**Published:** 2025-05-29

**Authors:** Katarzyna Unrug-Bielawska, Zuzanna Sandowska-Markiewicz, Kazimiera Pyśniak, Magdalena Piątkowska, Paweł Czarnowski, Krzysztof Goryca, Andrzej Mróz, Natalia Żeber-Lubecka, Urszula Wójcik-Trechcińska, Aneta Bałabas, Michalina Dąbrowska, Piotr Surynt, Mariusz Radkiewicz, Michał Mikula, Jerzy Ostrowski

**Affiliations:** 1 Department of Genetics, Maria Sklodowska-Curie National Research Institute of Oncology, Roentgena 5, Warsaw, 02-781 Poland; 2 Department of Gastroenterology, Hepatology and Clinical Oncology, Centre of Postgraduate Medical Education, Warsaw, Poland; 3 Department of Pathology, M. Sklodowska-Curie National Research Institute of Oncology, Warsaw, Poland; 4 Mass Spectrometry Laboratory, Institute of Biochemistry and Biophysics, Polish Academy of Sciences, Warsaw, Poland

**Keywords:** Western Diet, Fecal microbiota transplantation (FMT), Mtarc2 knockout mice, Stool microbiota, Stool metabolites

## Abstract

**Background:**

The mitochondrial amidoxime-reducing component-2 (Mtarc) enzyme complex is located on the outer mitochondrial membrane and may be involved in lipid metabolism regulation.

**Aim:**

This study evaluated the impact of fecal microbiota transplantation (FMT) on phenotypic outcomes, liver accumulation of fatty acids (FAs), and modifications to the gut microbial community, as well as the abundance of short-chain fatty acids (SCFAs) and amino acids (AAs), in both sexes of Mtarc2 knockout (Mtarc2-KO) and C57BL/6 N mice fed a Western Diet (WD).

**Methodology:**

Mice were fed a WD (study groups) or normal diet (control groups) and were subjected to intestinal flushing with either a polyethylene glycol (PEG) solution (study groups) or water (control groups); this was followed by intragastrical administration of a human feces suspension (study groups) or water (control groups). Liver FA composition and fecal SCFAs and AAs were measured by mass spectrometry. Metagenomic-based analysis was performed by sequencing the variable V3 and V4 regions of the bacterial 16 S rRNA gene.

**Principal findings:**

Weight gain in C57BL/6 N mice fed a WD was significantly higher than in Mtarc2-KO mice. Compared with water only, intestinal cleansing with PEG resulted in significantly lower weight gain in C57BL/6 N mice but not in Mtarc2-KO mice. FMT did not affect body weight in C57BL/6 N mice, and decreased in Mtarc2-KO females and males fed a ND and a WD, respectively. No significant differences in liver FAs composition were found between mouse strains. While PEG treatment significantly affected liver FAs composition, FMT modulated FAs levels to a much smaller extent. However, neither intestinal cleansing nor FMT affected the microscopic findings of fatty liver. WD feeding affected bacterial diversity, taxonomy and SCFAs and AAs abundances in Mtarc2-KO and C57BL/6 N mice not subjected to PEG treatment. Both intestinal cleansing alone and FMT modulated gut bacterial composition, especially in C57BL/6 N mice, and metabolite abundances in Mtarc2-KO mice.

**Conclusion:**

WD and FMT differentially modified phenotypic parameters, liver FA composition, and gut bacteria in comparisons between Mtarc2-KO and C57BL/6 N. This suggests the Mtarc complex plays a significant role in regulating energy metabolism in mice.

**Supplementary Information:**

The online version contains supplementary material available at 10.1186/s12263-025-00772-x.

## Background

A healthy diet offers essential nutrition that promotes a “healthy” gut microbiome, while high-fat or high-sugar diets can affect intestinal microbial compositions in a way that is linked with obesity [1]. The gut microbiota harvests nutrients and energy from the diet and produces metabolites with local and systemic actions, such as short-chain fatty acids (SCFAs), amino acids (AAs), secondary bile acids, and lipopolysaccharides, which train the host’s immune system and protects against opportunistic pathogens [2]. By modulating enterocyte-produced paracrine signaling molecules, including the YY peptide, ghrelin, insulin, glucagon-like peptide-1 (GLP-1), and the formation of specific bile acids [3], the gut microbiota can affect food intake and satiety; by contrast, dysbiosis affects appetite and hedonic aspects of food intake, energy absorption, fat storage, and circadian rhythm through a complex network of host–microbe interactions [4, 5]. As a consequence, gut microbiota is considered an important environmental factor contributing to obesity, it is also linked to adiposity, diabetes, and dyslipidemia [1, 6]. In addition, animal studies indicate that host phenotypes can be affected by fecal microbiota transplantation (FMT) [7]. For example, FMT from mice with obesity, either because of genetic or diet-related reasons, into normal-weight mice increases the body weight of lean mice; in addition, when mice lacking gut microbiota receive an FMT from either an obese and normal-weight individual, they adopt the weight-related phenotype of the donor [8–10].

Some bacteria, including *Bacteroides*, *Roseburia*, *Bifidobacterium*, *Faecalibacterium*, and *Enterobacteriaceae*, facilitate the extraction of calories from the diet and provide energy and nutrients for bacterial growth and proliferation. They typically ferment undigested carbohydrates and SCFAs (namely butyrate, propionate, and acetate) [11, 12]. Propionate and acetate are primary substrates for liver lipogenesis and gluconeogenesis, the latter of which provides energy for colonic epithelial cells. SCFAs are also involved in the regulation of gene expression by binding to the G-protein-coupled receptors 41 and 43, with several potential outcomes, including increased nutrient absorption. Furthermore, SCFAs are used as energy sources in different tissues: acetate enters the systemic circulation and reaches peripheral tissues; propionate is used mainly in the liver; and butyrate is used in the intestinal epithelium [13]. Species belonging to the Firmicutes and Actinobacteria phyla are producers of conjugated linoleic acid [14]. Conjugated linoleic acid can remove arachidonic acid from cell membrane phospholipids and may mediate the activation of transcription factors, such as peroxisome proliferator-activated receptors (PPARs), which are involved in lipid metabolism, apoptosis, and immune function [15].

Intestinal microbiota may affect intestinal lipid absorption, thus contributing to the development of obesity [16]. Obesity is associated with fat accumulation in the liver, which results from an imbalance between hepatic uptake and export of fatty acids (FAs), de novo lipogenesis, and fat utilization by β-oxidation [17]. Hepatic lipid uptake is controlled by fatty acid transport proteins and cluster of differentiation 36, the levels of which increase in the liver in response to Western Diet (WD) feeding [18]. De novo lipogenesis is regulated in the liver by three enzymes: acetyl-CoA carboxylase, fatty acid synthase, and stearoyl-CoA desaturase-1. Newly generated FAs undergo biological modifications, such as desaturation, elongation, and esterification, before being stored as triglycerides or exported as very low-density lipoprotein cholesterol (LDL-C) particles [19]. Oxidation of FAs primarily occurs in the mitochondria, and FAs entry into mitochondria depends on carnitine palmitoyltransferase 1, which is regulated by PPARα [19–22]. Although the obesity phenotype can be reversed by modulating gut microbiota, relationships between gut microbiota and lipid metabolism related to obesity are still poorly defined.

A WD results in obesity, which is accompanied by alterations in the hosts’ energy metabolism, immune system, and gut barrier function that occur because of imbalanced gut microbiota and increased intestinal permeability [15, 17]. However, a WD can impact the gut microbiota irrespective of whether mice develop obesity; this raises the question of whether there is a causal relationship between intestinal dysbiosis and obesity [23].

The mitochondrial amidoxime–reducing component (Mtarc) is one of five known molybdenum enzymes in eukaryotes. Its two paralogues, Mtarc1 and Mtarc2, together with cytochrome b5b (Cyb5b) and NADPH-dependent cytochrome b5b reductase (Cyb5R), form an enzymatic complex that reduces a large variety of N-hydroxylated compounds [19, 20]. However, a potential connection between energy homeostasis, particularly lipid metabolism and Mtarc enzymes has also been demonstrated [24]. While the relationship between changes in the gut microbiota and energetic homeostasis has mainly been studied in humans with overweight/obesity or in animal models of obesity, in this study, we determined how a WD and repeated FMT from human donors affected phenotype, accumulation of liver FAs, and modifications of the gut microbial community, as well as SCFAs and AAs abundance, in Mtarc2 knockout mice (Mtarc2-KO) compared with background C57BL/6 N mice. Mtarc2-KO mice gained significantly less body weight than C57BL/6 N mice on a normal diet (ND), an effect that was even more pronounced on a high fat diet [25]. These results indicate the involvement of the Mtarc2 protein in lipid metabolism.

## Materials and methods

### Mice

The Local Ethics Committee approved experimental procedures for animal testing (decisions: WAW2/119/2019 and WAW2/124/2021). The studies were carried out according to the European Parliament and the Council Directive (2010/63/EU) and the Polish regulations on the protection of animals used for scientific and educational purposes (Journal of Laws 2021, items 1331 and 2338).

Mtarc2-KO mice and background strain C57BL/6 N mice were born without specific pathogens at the Maria Sklodowska-Curie National Research Institute of Oncology, Warsaw. Mice were kept in standard humidity (55% ± 10%) and temperature (21 ± 2 °C) conditions in climate-controlled rooms under a 12 h light/dark cycle. Animals were tested for the presence of viruses, bacteria, and parasites according to the recommendations of the Federation of European Laboratory Animal Science Associations.

One hundred and four 6–8-week-old C57BL/6 N mice and 104 6–8-week-old Mtarc2-KO mice of both sexes were allowed to adapt to the experimental facility for 2 weeks. They were then randomly divided into groups comprising 6–10 mice per group. Half of these groups received an ND containing 4.4 g fat, 16.5 g protein, and 70.6 g carbohydrate in 100 g dry weight feed, with a metabolic energy content of 16.38036 MJ(RD Western Diet D14042701N; Research Diets, Inc., New Brunswick, NJ, USA). The remaining mice were fed an WD containing 21 g fat, 19.8 g protein, and 49.9 g carbohydrate in 100 g dry weight feed, with a metabolic energy content of 19.576936 MJ (RD Western Diet D12079B; Research Diets, Inc.). Mice were housed 3–4 in a cage with a 12-hour light/dark cycle, with unrestricted access to water and food throughout the experiment.

Of the experimental mice, 72 Mtarc2-KO mice and 72 C57BL/6 N mice of both sexes received FMT. On the day of the first FMT, mice were transferred to clean cages to avoid coprophagia and were deprived of access to food for 1 h. Then, mice were assigned randomly into the study and control groups. Mice from the study group were subjected to intestinal flushing with four oral gastric gavages containing 200 µL of an aqueous solution of Macrogol 4000 (Ipsen Consumer HealthCare) (polyethylene glycol, PEG) at a concentration of 425 g/L, which were given at 20-minute intervals [the PEG(+) groups]. Control mice received four oral gastric gavages with 200 µL of water [the PEG(−) groups]. Four hours after the last intragastrical administration of PEG, the mice did (FMT) or did not (NFMT) undergo a human stool transplantation procedure by receiving 200 µL of human feces suspension from lean or obese individuals, or an aqua pro injection given intragastrically to the study and control mice, respectively. The FMT procedure was carried out at 16 once-weekly intervals, under short anesthesia using isoflurane.

To prepare fecal suspensions, one gram of stool sample from each of 3 healthy lean individuals (two women and one man; BMI ≤ 25 kg/m^2^) and from each of 3 individuals with obesity (two woman and one man; BMI 36, 38, and 42 kg/m^2^) was pooled and suspended in PBS (1 g stool/5 ml PBS). The suspension was homogenized and centrifuged (1500 rpm), the supernatants were filtered through 100 μm Millipore filters, glycerol was added to the filtrate to a final concentration of 10%, aliquoted and stored at -80 °C. Immediately before administration to mice, the suspension was diluted 4-fold with sterile water. Sequencing of bacterial 16 S rRNA gene isolated from individual donor samples showed distinct microbiota composition as revealed by α- and β-diversity and taxonomic analyses (Additional Fig. 1).

**Fig. 1:**
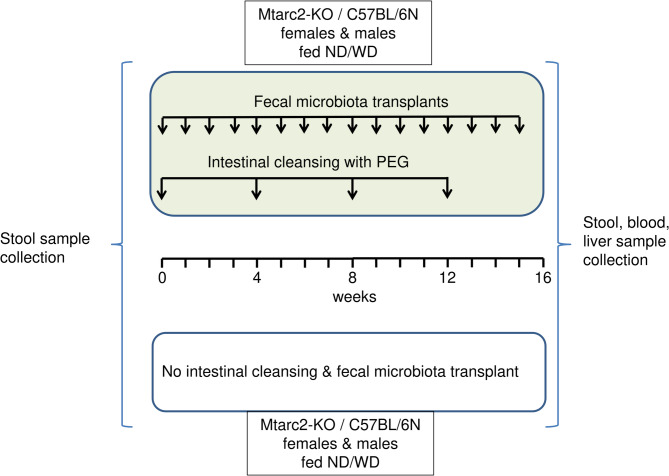
Graphical summary of the study design. The study was conducted in mitochondrial amidoxime-reducing component-2 knockout mice (Mtarc2-KO) and C57BL/6 N mice of both sexes. Mice were fed a Western Diet (WD; study groups) or normal diet (ND; control groups) and were subjected to an oral gavage of polyethylene glycol (PEG; study groups) or water (control groups). In fecal matter transplantation (FMT) mice, on the first day of the experiment (time 0; T0), intestinal cleansing with the PEG solution and administration of the pooled stool extracts from lean or obese humans were performed by oral gavage. Intestinal cleansing was repeated four times at 4-week intervals, and FMT was repeated 16 times weekly. The groups consisted of 6–10 mice. Stool samples were collected at T0 and the end of the experiment (T16); blood and liver tissue samples were collected at T16. The collected samples were frozen at − 80 °C

At the end of the experiment, mice were anesthetized with 5% isoflurane and sacrificed by cervical dislocation; blood, liver, and visceral and gonadal fat were immediately acquired. Stool samples were collected from each mouse before and at the end of the experiment and stored at − 80 °C until use. The experimental procedure used in this study is illustrated in Fig. 1.

### Serum biochemical measurements

Serum cholesterol, HDL-C, triglyceride, alanine aminotransferase, aspartate aminotransferase, and alkaline phosphatase levels were measured using an automated clinical chemistry analyzer (Spotchem EZ SP-4430; ARKRAY, Kyoto, Japan) and the corresponding test strips.

### Histological analysis

A freshly collected liver tissue sample measuring 1 cm^2^ was washed with saline, immediately fixed in 4% paraformaldehyde solution, and stained with hematoxylin and eosin to histologically evaluate steatosis, inflammation, and hepatocellular ballooning according to the Histological Scoring System for Nonalcoholic Fatty Liver Disease [26].

### Liver fatty acids analysis

Standards of 18 fatty acids (Caprylic acid, Decanoic acid, Lauric acid, Myristic acid, Linoleic acid, α-Linolenic acid, γ-Linolenic acid, Trans-vaccenic acid, Arachidic acid, Eicosapentaenoic acid, Arachidonic acid, Dihomo-γ-linolenic acid, Adrenic acid, Erucic acid, Docosahexaenoic acid, Nervonic acid, Oleic acid and Palmitoleic acid) and isotopically labeled analogous (Decanoic acid – 1,2 C13, Myristic acid – 1,2 C13, Stearic acid − 17,17,18,18,18 - d5, Eicosapentaenoic acid − 19,19,20,20,20 - d5, Arachidonic acid − 5,6,8,9,11,12,14,15 - d8, Docosahexaenoic acid − 21,21,22,22,22 – d5) were purchased from Sigma Aldrich (Merck).

Mouse liver samples were weighed using an analytical balance. Liver fragments were transferred to homogenization tubes filled with porcelain beads. 9 µL of HPLC grade ethanol was added per 1 mg of weighed tissue. The samples were homogenized with a Precellys^®^ Evolution Touch orbital homogenizer for 10 min at 4500 RPM at 4 ° C.

25 µL of homogenate was aspirated into a new sample tube. 125 µL of internal standard (5 µg/mL of each isotopically labeled analogue) in HPLC grade acetonitrile was added. Samples were vortexed for 1 min at 1,500 RPM. 20 µL of 6 N HCl was added and boiled at 104 ° C for 45 min. Next, 20 µL of 10 N NaOH was added and boiled at 104 ° C for 45 min. Subsequently, 100 µL of 6 N HCl was added to all samples and they were vortexed for 1 min at 1,500 RPM. Liquid-liquid extraction was achieved by adding 600 µL of HPLC grade hexane, vortexing for 10 min at 1,500 RPM and spinning for 10 min at 14,000 RPM. The lipophilic phase was transferred to a new sample tube and evaporated to dryness under a nitrogen stream. The lipids were reconstituted in 100 µL of 65% HPLC grade methanol with 5% NH4OH and subjected to LC-MS analysis.

LC-MS analysis was performed using the Acquity UPLC system (Waters) coupled with the Xevo TQ mass spectrometer (Waters). Fatty acids were separated using the BEH C18 column (2.1 × 100 mm 1.7 μm, Waters) heated to 50 °C. The binary gradient of mobile phase A (0.1% NH4OH in MQ grade water) and mobile phase B (LC-MS grade acetonitrile) started at 20% phase B and increased to 95% in 2.8 min. The total run time was 4.0 min. The mass spectrometer operated in negative ionization mode (ESI-) and spectra were acquired using multiple reaction monitoring mode (MRM).

### 16s-rRNA-Seq metagenomics and metabolomics procedures

Genomic DNA was isolated from fecal samples using a QIAamp Fast DNA Stool Mini Kit (Qiagen, Hilden, Germany) according to the manufacturer’s protocol, as previously described [27]. The quality and quantity of the extracted DNA was assessed by measuring the optical density using a NanoDrop 2000/2000c spectrophotometer (Thermo Fisher Scientific, Carlsbad, CA, USA) and fluorometrically using a Qubit dsDNA HS Assay Kit (Thermo Fisher Scientific). Library preparation of the variable V3 and V4 regions of the 16 S bacterial 16 S rRNA gene was carried out according to the 16 S Metagenomic Sequencing Library Preparation protocol on an Illumina platform (Illumina, Inc., San Diego, CA, USA). Sequences were obtained on an Illumina MiSeq system in a 2 × 300 bp paired-end run.

SCFAs and AAs were extracted and derivatized as previously described [2, 27]. Gas chromatographic analysis of fecal extracts was performed on an Agilent 7000D triple quadrupole mass spectrometer coupled to a 7890 gas chromatography system with a G4513A autosampler (Agilent Technologies, Santa Clara, CA, USA). A VF-5ms column (30 m, 0.25 mm, 0.50 μm) was used for the analysis. Mass spectrometry data were collected in full-scan mode for m/z 15–650 at a frequency of 4.9 scans per second. MassHunter software (Agilent Technologies) was used for the analysis.

### Statistical methods

The DADA2 [28] pipeline version 1.30 was used for read error correction, amplicon sequence variant identification, and chimeric read identification and removal. Taxonomy assignment was carried out with DECIPHER version 2.30 [29] using SILVA (version 1.138 [30]). Diversity indexes (Shannon and Chao1) were calculated with the phyloseq R package version 1.46 [31]. Significant differences between groups were identified with the Deseq2 package [32]. Distances (β-diversity) were calculated with the phyloseq package [31] using the Euclidean metric. Differences in the relative abundances of AA and SCFAs were identified with the Mann–Whitney U test or mixed-effects linear models.

## Results

### Body weight

**Fig. 2:**
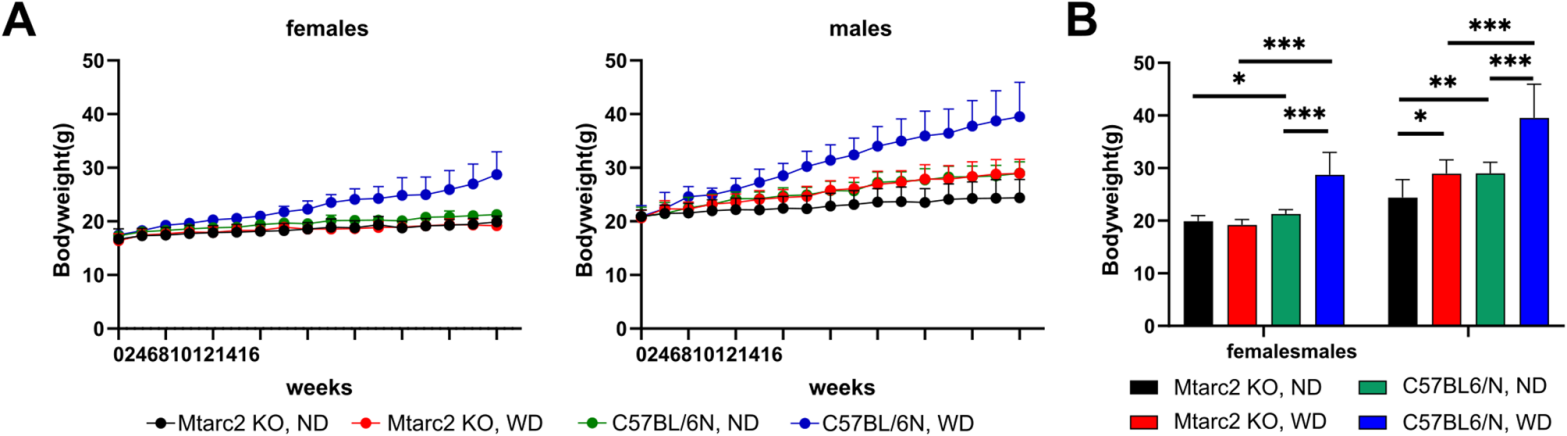
**A** Evaluation of body weight Mtarc2 KO and C57BL6/N female and male groups in a 16-week experiment; **B** Body weight gain at the endpoint of a 16-week experiment between groups of mice that were not subjected to the polyethylene glycol (PEG) treatment. Values are expressed as mean ± SD, *n* = 8–10 mice per group, and statistical assessment is conducted using a three-way ANOVA; * *p* < 0.05, ** *p* < 0.01 *** *p* < 0.001

The animals were fed an ND or a WD for 16 weeks. Comparison between ND and WD-fed mice from PEG(−) groups revealed that WD feeding resulted in higher body weights in male and female C57BL/6 N NFMT mice, a difference that increased over the course of the experiment; this effect was present to a much lower extent in Mtarc2-KO mice (Fig. 2A). To determine how the body weight at the end of the experiments was affected by diet, sex and genotype, a three-way ANOVA was conducted. As shown in Fig. 2B, the body weight of both sexes of ND-fed mice did not differ between strains. WD feeding resulted in significantly higher body weight gain in C57BL/6 N mice of both sexes (*P* < 0.001) but not in Mtarc2-KO mice. Weight gain in C57BL/6 N mice was significantly higher than in Mtarc2-KO mice fed a WD (Fig. 2B). With the exception of ND-fed Mtarc2-KO mice, in the remaining three groups of mice, the body weight of males was significantly higher than that of females at the end of the experiments.

Intestinal flushing with PEG, which was performed four times at monthly intervals, did not affect body weight in either female or male Mtarc2-KO mice fed an ND or a WD (Additional Fig. 2A). By contrast, weight gain in C57BL/6 N male mice fed ND and in both females and males fed WD was significantly lower in PEG(+) groups than in PEG(−) groups (Additional Fig. 2B). Compared to mice that were subjected only to intestinal flushing with PEG, Mtarc2-KO female mice fed ND and Mtarc2-KO male mice fed WD that had undergone FMT with feces from lean and obese human donors had significantly lower weight gain (Additional Fig. 2C). No effect of FMT on body weight was observed in C57BL/6 N mice (Additional Fig. 2D).

### Weights of the liver, visceral fat, and gonadal fat and serum lipid levels

In Mtarc2-KO females, 16-week feeding with WD did not affect liver weight (Additional Fig. 3A); however, the weights of visceral (Additional Fig. 3B) and gonadal (Additional Fig. 3C) fat were significantly lower than in ND-fed mice. In Mtarc2-KO males and C57BL/6 N mice of both sexes fed a WD, all organ/tissue weights were significantly higher than in ND-fed mice (Additional Fig. 3A–C). In addition, compared with ND-fed mice, WD feeding resulted in significantly higher serum concentrations of total cholesterol and LDL-C in all mice (Additional Fig. 3D and E), as well as triglyceride concentrations only in the C57BL/6 N female group (Additional Fig. 3F).

**Fig. 3:**
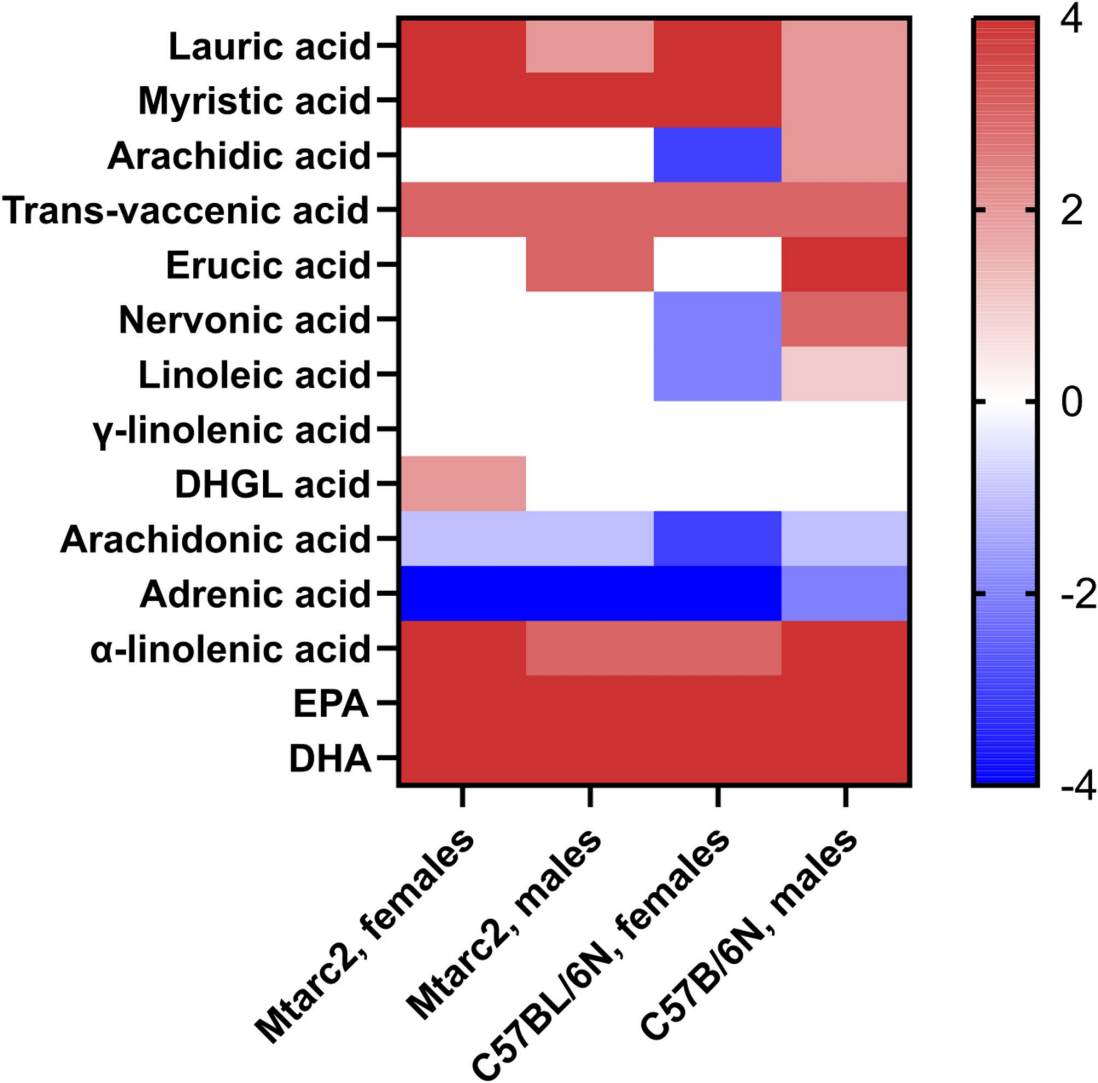
Heatmap of differences in liver fatty acid concentrations in Mtarc2-KO and C57BL/6 N mice fed a Western Diet (WD) compared to concentrations found in mice on a normal diet (ND) not subjected to intestinal cleansing

While most phenotypic and biochemical measures were not affected by intestinal flushing with PEG (Additional Figs. 4 and 5), compared with PEG(−) mice, liver weight was significantly higher in C57BL/6 N males fed ND (Additional Fig. 4A) and in Mtarc2-KO females fed WD (Additional Fig. 4D); in addition, serum concentrations of HDL-C and triglycerides in ND-fed females of both strains were significantly higher in PEG(+) mice than in PEG(−) mice (Additional Fig. 5A and B).

Similarly weak effects resulted from FMT compared with mice that were subjected only to intestinal cleansing (NFMT mice), those that received an FMT from lean or obese human donors had significantly higher liver weights (WD-fed Mtarc2-KO females) (Additional Fig. 6A), significantly higher gonadal fat weights (ND-fed Mtarc2-KO females) (Additional Fig. 6E), and significantly higher serum total cholesterol (WD-fed C57BL/6 N males) (Additional Fig. 7C). WD-fed Mtarc2-KO males that had undergone an FMT from lean donors had significantly lower liver weights than NFMT mice (Additional Fig. 6A), and ND-fed C57BL/6 N males that had undergone an FMT from obese donors had significantly higher serum total cholesterol than NFMT mice (Additional Fig. 7B).

### Liver histology

At the end of the experiment, no fatty liver was observed in any PEG(−) mice fed an ND, while mixed micro and macrovesicular steatosis was found) (Additional Fig. 8), mainly around the portal area, in almost all liver sections in WD-fed mice (Table 1). However, the intensity of steatosis differed between groups. Steatosis reached grade 3 in eight out of 10 C57BL/6 N males and only two out of 10 Mtarc2-KO males fed the WD. Grade 1 liver steatosis was found in all seven Mtarc2-KO females and five out of the eight C57BL/6 N females fed the WD. Lobular and portal inflammation that reached histological grades 1 or 2 was present in more than half of Mtarc2-KO mice, independent of sex and diet, and in C57BL/6 N males, while grade 1 inflammation was found in only four out of 10 female C57BL/6 N WD-fed mice. Hepatocyte ballooning was observed in both Mtarc2-KO and C57BL/6 N mice fed the WD, with a higher intensity in males (Table 1).

**Table 1:** Effects of feeding a normal diet (ND) or a Western diet (WD) for 16 weeks in mitochondrial amidoxime-reducing component-2 knockout mice (Mtarc2-KO) (A) and C57BL/6 N (B) mice from polyethylene glycol [PEG(−)] groups on liver steatosis, liver lobular inflammation, and liver cell balloon injury according to the Non-Alcoholic fatty liver disease activity score [26]

Mice	Diet/ Sex	Steatosis				Lobular inflammation				Ballooning		
		Scores										
		0	1	2	3	0	1	2	3	0	1	2
		Number of samples classified according to the scoring system:										
mARC2-KO	ND/F	7				2	2	3		7		
	ND/M	9				3	3	3		9		
	WD/F		7			1	4	2		5	2	
	WD/M		6	2	2	5	3	3		5	1	4
C57BL6/W	ND/F	8				9				9		
	ND/M	9				9				9		
	WD/F	1	5	1	1	2	5	1		5	3	
	WD/M		1	1	8	6	4				5	5

Steatosis scoring system: 0, < 5%; 1, 5–33%; 2, 34–66%; 3, > 66%; lobular inflammation scoring system: 0, no foci; 1, < 2 foci per 200× field; 2, 2–4 foci per 200× field; 3, > 4 foci per 200× field; hepatocellular ballooning scoring system: 0, none; 1, few cells with ballooning; 2, many cells with prominent ballooning

*ND* normal diet, *WD* Western diet

Similar to PEG(−) mice, WD feeding in PEG(+) mice resulted in fatty liver, which was slightly more severe in males than in females, especially in C57BL/6 N mice. Some but weak portal and lobular inflammation were observed in mice from almost all groups, independent of mouse strain, sex, and diet. As summarized in Table 2, FMT did not affect microscopic findings in the experimental groups.

**Table 2:** Effects of intestinal cleansing with PEG and fecal microbiota transplantation on liver steatosis, liver lobular inflammation, and liver cell balloon injury according to the Non-Alcoholic fatty liver disease activity score [33]

Mice	Diet/ Sex	FMT	Streatosis				Lobular inflammation				Ballooning		
			Scores										
			0	1	2	3	0	1	2	3	0	1	2
			Number of samples classified according to the scoring system:										
Mtarc2-KO	ND/F	(-)	5				4	1			2	2	1
	ND/M	(-)	5				2	3			2	1	2
	WD/F	(-)	1	4				4	1		1	3	1
	WD/M	(-)		1	2	2		5			1	1	3
	ND/F	Lean	5				3	2			2	1	2
	ND/M	Lean	5				2	3			1	1	3
	WD/F	Lean		5			2	3			1	2	2
	WD/M	Lean	1	3	1		3	2				3	2
	ND/F	Obese	3	2			2	2	1		3	1	1
	ND/F	Obese	4	1			5				1	1	3
	WD/F	Obese		4				2	2		2	1	1
	WD/M	Obese		3	1	1	3	2			1	2	2
C57BL/6N	ND/F	(-)	4	1			1	4			2	2	1
	ND/M	(-)	5				3	2			2	2	1
	WD/F	(-)		3	2	1		4	2		2	4	
	WD/M	(-)			1	5	1	3	2		4	1	1
	ND/F	Lean	2	3			1	4				2	2
	ND/M	Lean	5				2	2	1			3	2
	WD/F	Lean		1	2	2		3	2		1	2	2
	WD/M	Lean			1	4	2	3			4	1	
	ND/F	Obese	3	2			3	2			1	2	2
	ND/M	Obese	4	1				4			1	2	2
	WD/F	Obese			2	3	1	4			1	3	1
	WD/M	Obese				5	3	2			2	2	1

(−), intestinal cleansing with polyethylene glycol (PEG) without FMT; lean, FMT with stool suspension prepared from lean humans; obese, FMT with stool suspension prepared from obese humans. Steatosis scoring system: 0, < 5%; 1, 5–33%; 2, 34–66%; 3, > 66%; lobular inflammation scoring system: 0, no foci; 1, < 2 foci per 200× field; 2, 2–4 foci per 200× field; 3, > 4 foci per 200× field; hepatocellular ballooning scoring system: 0, none; 1, few cells with ballooning; 2, many cells with prominent ballooning

*F* female, *M* male, *ND* normal diet, *WD* Western diet, *FMT* fecal microbiota transplantation

### Hepatic fatty acid levels

Concentrations of individual hepatic FAs, including lauric acid (C12:0), myristic acid (C14:0), and arachidic acid (C20:0) (saturated FAs); trans-vaccenic acid [trans11-(18:1)], erucic acid (C22:1n9), and nervonic acid (C24:1n9) (monounsaturated FAs); linoleic acid (C18:2n6), γ-linolenic acid (C18:3n6), di-homo-γ-linolenic acid (C20:3n6), arachidonic acid (C20:4n6), and adrenic acid (C22:4n6) (n-6 polyunsaturated FAs); and α-linolenic acid (C18:3n3) and eicosapentaenoic acid (C20:5n3) (n-3 polyunsaturated FAs) ranged from microgram to milligram amounts per gram of liver tissue. Although the general FA pattern was similar between Mtarc2-KO and C57BL/6 N mice fed an ND without intestinal cleansing, there were some differences between the strains (Table 3).

**Table 3:** Comparisons of basic hepatic fatty acid concentrations (µg/g of liver sample) between Mtarc2-KO and C57BL/6 N females and males fed a normal diet (ND) for 16 weeks

Fatty acid	Females, normal diet	Females, high fat diet	Males, normal diet	Males, high fat diet
	Mean of Mtarc2-KO vs. mean of C57BL/6 *N*	Mean of Mtarc2-KO vs. mean of C57BL/6 *N*	Mean of Mtarc2-KO vs. mean of C57BL/6 *N*	Mean of Mtarc2-KO vs. mean of C57BL/6 *N*
Lauric acid	0 vs. 0	33,29 vs. 46,18	3,29 vs. 0^*^	100,9 vs. 141
Myristic acid	141,7 vs. 161	470,2 vs. 715,9^*^	165,2 vs. 172,6	1566 vs. 917,9^*^
Arachidic acid	3,1 vs. 72,9^***^	0 vs. 35,63^***^	115,6 vs. 159,9	257,3 vs. 449,9
Trans-vaccenic acid	1695 vs. 2119	3586 vs. 4443	1763 vs. 1852	4196 vs. 6499
Erucic acid	0 vs. 0	3,87 vs. 0^*^	24,7 vs. 39,77	115,8 vs. 951,9^****^
Nervonic acid	114 vs. 143,3	98,02 vs. 100,4	121,2 vs. 76,04^*^	112,3 vs. 212,4^**^
Linoleic acid	4164 vs. 5469	3366 vs. 3080	3974 vs. 3992	3790 vs. 5323^**^
γ-linolenic acid	43,7 vs. 63,9^*^	54,33 vs. 59,04	31,48	48,24 vs. 63,03
di-homo-gamma-linolenic acid	461,1 vs. 494,1	654,1 vs. 563,5	714,2 vs. 724,5	667,2 vs. 901,4
Arachidonic acid	5844 vs. 7562	4672 vs. 3801	5391 vs. 5798	4149 vs. 4107
Adrenic acid	378,1 vs. 482,8^**^	238,9 vs. 218,7	415,2 vs. 391,1	257,3 vs. 449,9
α-linolenic acid	0 vs. 17,5^****^	47,79 vs. 57,75	14,79 vs. 6,32	64,5 vs. 116,3^**^
Eicosapentaenoic acid	10,4 vs. 4,6	207,5 vs. 173	4,64 vs. 17,83^***^	173,6 vs. 150,3
Docosahexaenoic acid	2691 vs. 3114^*^	5474 vs. 4661^*^	2629 vs. 2089^**^	4376 vs. 4532

^*^p < 0.05

^**^p < 0.01

^***^p < 0.001

^****^p < 0.0001

Compared with ND, WD feeding resulted in significantly higher levels of lauric acid, myristic acid, trans-vaccenic acid, α-linolenic acid, eicosapentaenoic acid, docosahexaenoic acid in all groups; significantly higher concentrations of erucic acid in Mtarc2-KO and C57BL/6 N males; and significantly lower concentrations of arachidonic acid and adrenic acid in all groups. The concentrations of arachidic acid and nervonic acid were significantly higher in males and significantly lower in female C57BL/6 N mice, and erucic acid was significantly higher in Mtarc2-KO and C57BL/6 N males, as summarized in Additional Table 1 and by the heatmap in Fig. 3.

**Fig. 4:**
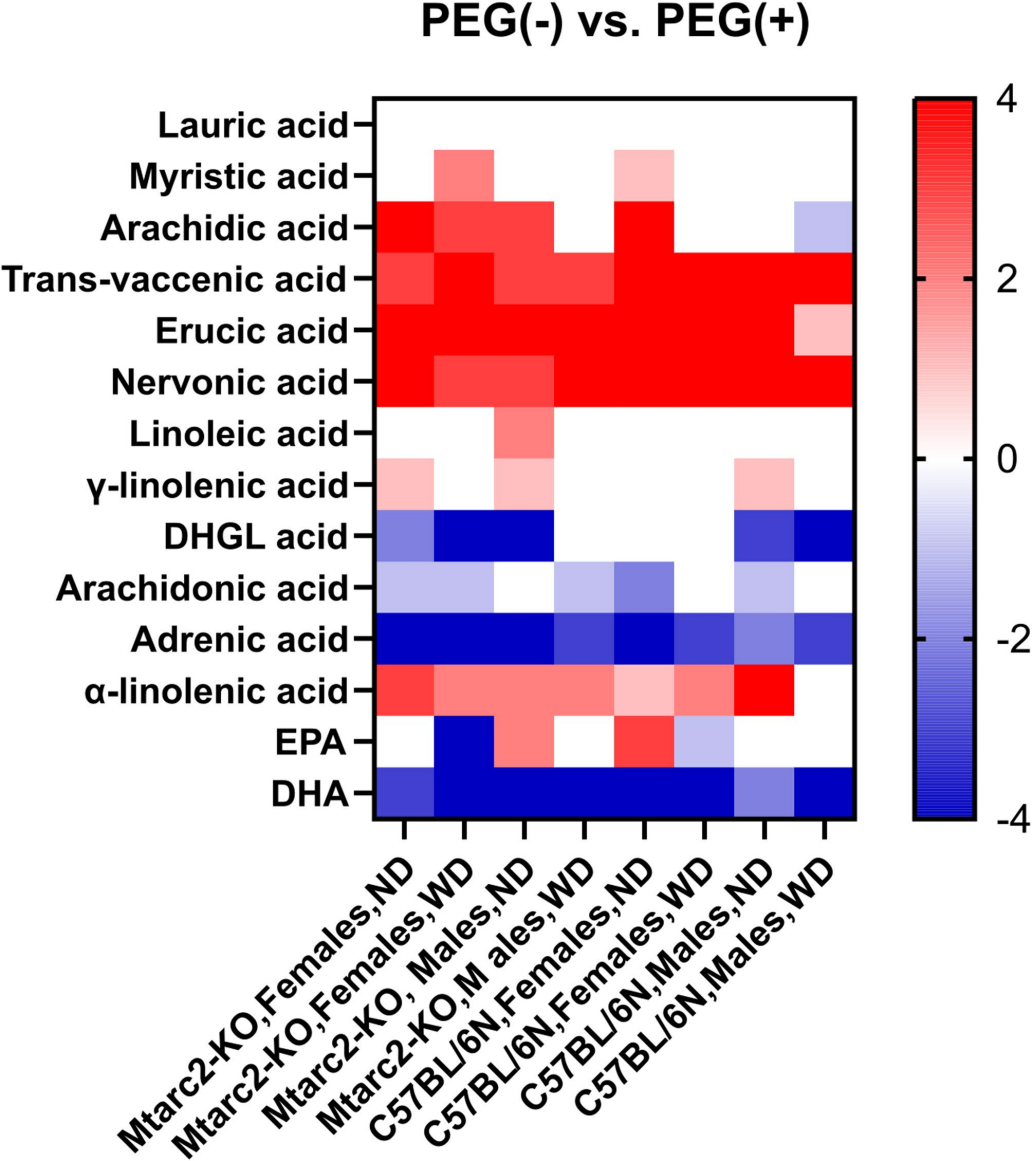
Heatmap of differences in hepatic fatty acid concentrations in Mtarc2-KO and C57BL/6 N mice treated with PEG compared to concentrations found in PEG-untreated mice

PEG administration significantly modified the levels of hepatic FAs (Additional Table 2). As summarized by the heatmap in Fig. 4, there were no significant differences in liver FAs concentration changes between mouse strains. Compared with the PEG(−) groups, intestinal flushing with PEG resulted in significantly higher levels of trans-vaccenic acid, erucic acid, nervonic acid, and α-linolenic acid in all or almost all experimental groups, independent of mouse strain, sex, and diet. By contrast, compared with the PEG(−) groups, significantly lower concentrations of adrenic acid and docosahexaenoic acid were found in each PEG(+) group, and significantly lower concentrations of di-homo-gamma-linolenic acid and arachidonic acid were observed in most of them. Lauric acid concentration was not influenced by treatment with PEG, and the remaining three FAs (myristic acid, linoleic acid, and γ-linolenic acid) were only affected by PEG treatment in specific groups without a clear pattern (Additional Fig. 9).

**Fig. 5:**
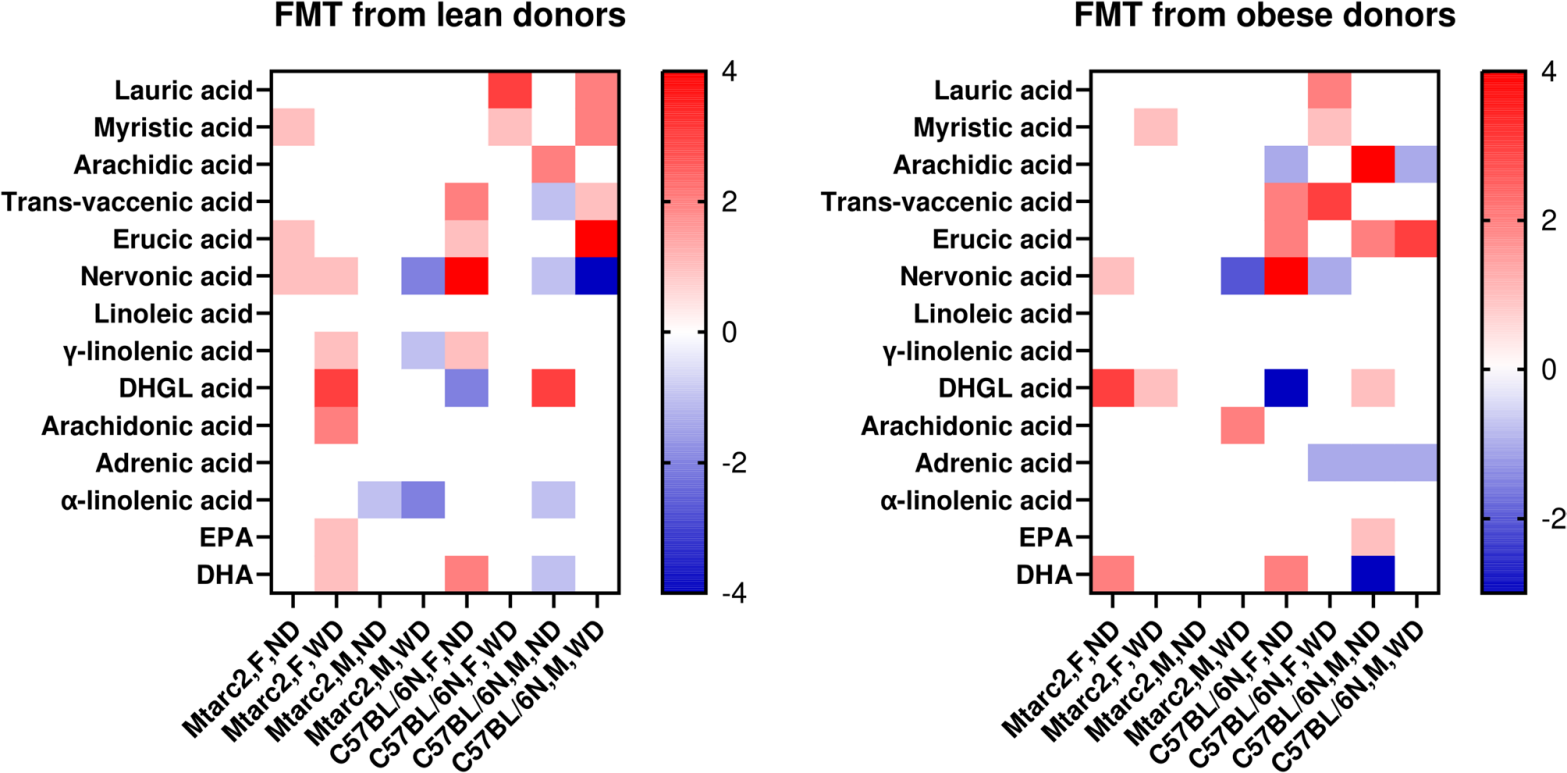
Heatmap of differences in hepatic fatty acid concentrations in Mtarc2-KO and C57BL/6 N mice treated that were only PEG treated [PEG/NFMT] and those transplanted with fecal microbiota isolated from lean [PEG/FMT(lean)] (left panel) of obese [PEG/FMT(obese)] (right panel) human donors

FMT further modulated FAs concentrations (Fig. 5 and Additional Fig. 9), although the observed effect was smaller than the impact of PEG treatment: there were more changes in PEG(+)/NFMT mice than in PEG(+)/FMT mice and this effect was stronger in C57BL/6 N mice than in Mtarc2-KO mice; there was no specific pattern of changes. However, it should be noted that experimental groups comprised only 5–6 mice and there was a relatively large variation in concentrations between individual FAs.

### Analyses of the gut bacterial community structure and metabolite profiles

Metagenomic-based analysis utilized sequencing of the variable V3 and V4 regions of the bacterial 16 S rRNA gene. An average of 75,616 reads were generated per sample (median, 77,007). Five of the nine identified phyla (*Firmicutes*,* Bacteroidota*,* Actinobacteriota*,* Verrucomicrobiota*, and *Desulfobacterota*) had an abundance equivalent to more than 1% of the microbiome. Of the 106 genera identified, 18 had an abundance equivalent to more than 1% of the microbiome; the five most prevalent were *Blautia*, *Alistipes*, *Akkermansia*, *Alloprevotella*, and *Bifidobacterium*.

The gut bacterial community structure was evaluated by analyzing the α- and β-diversity of fecal microbiota. α-diversity was analyzed using the Shannon index, a marker of bacterial richness and evenness, and the Chao1 index, a marker of bacterial richness. β-diversity was analyzed using principal coordinate analysis of Euclidean distances. Analyses were performed at the genus level. After multiple hypothesis testing corrections, the Shannon and Chao1 indexes did not differentiate females from males within a given strain or between strains at the beginning of experiments (Additional Fig. 10).

**Fig. 6:**
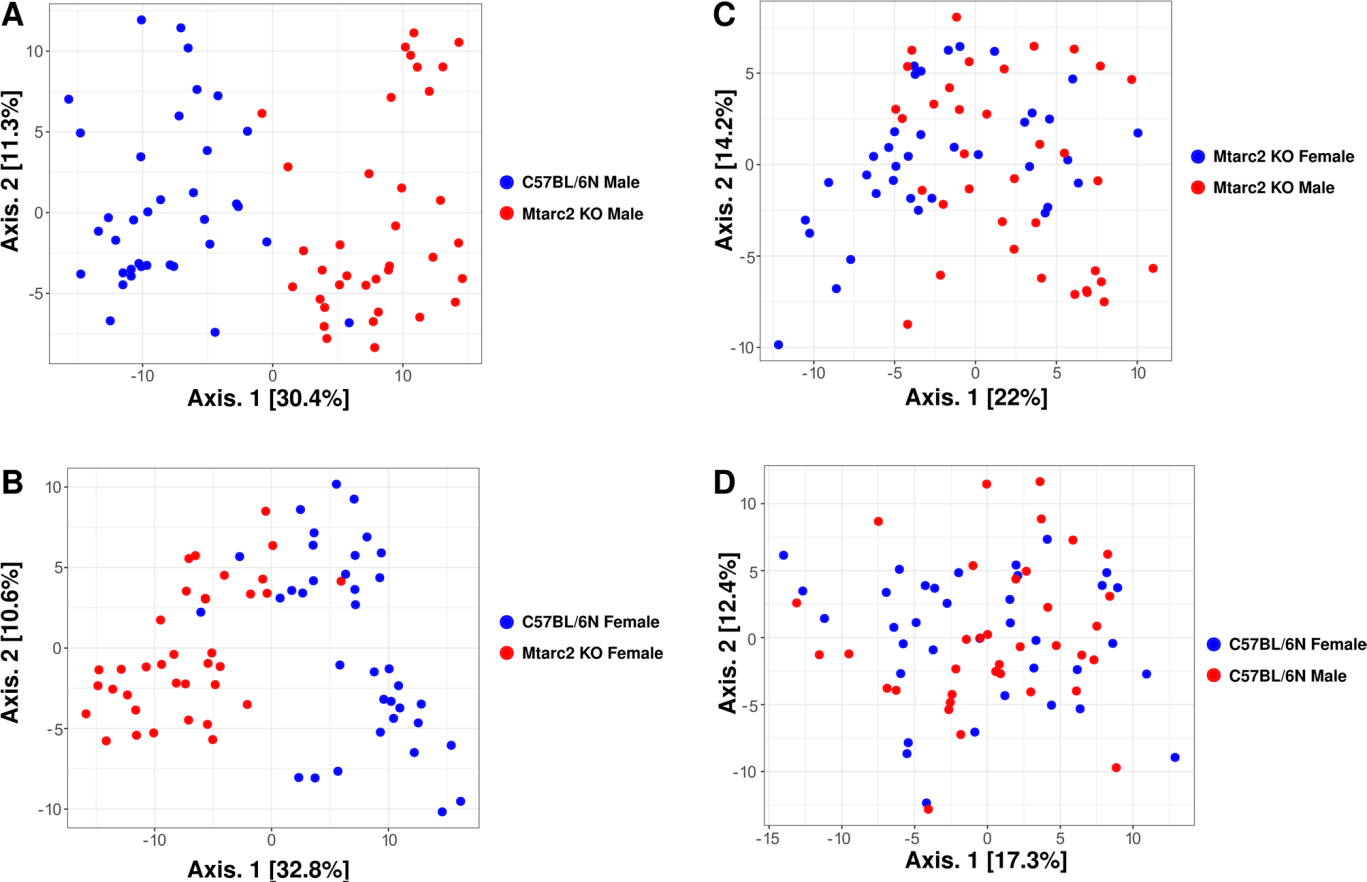
Principal coordinate analysis (PCoA) using the Euclidean metric of fecal samples collected at the beginning (T0) of experiments. Each dot represents a single sample

Instead, analyses of β-diversity showed significant differences between the Mtarc2-KO and C57BL/6 N groups, with p-values for the first component being 3.22E − 19 and 5.92E − 25 when comparing females and males, respectively (Fig. 6A and B). A significant difference was also found when comparing the Mtarc2-KO females and males, with p-values of 5.64E − 04 for the first component, but not when comparing C57BL/6 N females and males (*p* = 6.06E − 01) (Fig. 6C and D).

Sixteen weeks of WD feeding did not change Shannon index values in both mouse strains compared to the beginning of experiments, and resulted in significantly lower Chao1 index values in females of both Mtarc2-KO and C57BL/6 N mice (Additional Fig. 11).

Instead, β-diversity, measured by principal coordinate analysis, was significantly different between T16 and T0 in each of WD groups, with p-values of 2.88E − 07, 3.72E − 08, 0.0098, and 0.031 in C57BL/6 N females and males and Mtarc2-KO females and males, respectively (Additional Fig. 12).

Intestinal flushing with PEG in mice fed a WD resulted in significantly lower Shannon index scores in C57BL/6 N females, and significantly higher Chao1 index scores in C57BL/6 N females and males and Mtarc2-KO males compared with the corresponding PEG(−) group mice (Additional Fig. 13).

FMT from lean human donors resulted in significantly higher Shannon and Chao1 index scores in WD-fed C57BL/6 N males than in NFMT WD-fed males, and FMT from obese donors resulted in significantly higher Shannon index scores in WD-fed C57BL/6 N females than in NFMT WD-fed females (Additional Fig. 14).

By contrast, the β-diversity of the gut bacterial community structure was affected by FMT from both lean and obese human donors in all mice, with statistical significance for the first and second components individually and combined (Table 4).

**Table 4:** Statistical significance of differences in β-diversity, measured by principal coordinate analysis (PCoA) for the first and second components in fecal samples collected from WD-fed mice not transplanted (NFMT) and transplanted with fecal extracts from lean human donors [FMT (lean)] and obese [FMT (obese)] at the end time point (T16)

			Statistical significance	
			Principal coordinate analysis components	
Strain	Sex	Comparison	The first	The second
Mtarc2-KO	Females	NFMT vs. FMT (lean)	3,65E-06	ns
		NFMT vs. FMT (obese)	0,050	0,015
	Males	NFMT vs. FMT (lean)	0,015	0,029
		NFMT vs. FMT (obese)	ns	2,91E-06
C57BL/6 N	Females	NFMT vs. FMT (lean)	0,0065	ns
		NFMT vs. FMT (obese)	0,063	0,013
	Males	NFMT vs. FMT (lean)	5,24E-06	ns
		NFMT vs. FMT (obese)	0,00041	ns

*ns* not significant

Taxonomic analysis of fecal samples collected at the beginning of experiments (time point T0) uncovered 20 genera (7 over- and 13 underrepresented) that were different in Mtarc2-KO females compared with C57BL/6 N females, and 21 genera (8 over- and 13 underrepresented) that were different in Mtarc2-KO males compared with C57BL/6 N males (adjusted *p* < 0.05, Additional Table 3). Of these, five overrepresented genera (*Bifidobacterium*,* Ileibacterium*,* [Eubacterium] xylanophilum group*,* Coriobacteriaceae UCG-002*,* Harryflintia*) and seven underrepresented genera (*Paludicola*,* Lachnospiraceae UCG-006*,* Lachnospiraceae NK4A136 group*,* Mucispirillum*,* Butyricicoccus*,* Lactococcus*,* Defluviitaleaceae UCG-011*) that differentiated between Mtarc2-KO and C57BL/6 N mice were common to both sexes.

Comparison between T16 and T0 in PEG(−) mice showed 2 and 3 taxa that were overrepresented and underrepresented (adjusted *p* < 0.05) at the end of the experiment in ND-fed Mtarc2-KO females, and 4 and 4 taxa that were overrepresented and underrepresented, respectively, in Mtarc2-KO males. Similar comparison in C57BL/6 N mice groups showed 4 and 10 taxa over- and underrepresented in females and 8 and 12 taxa in males, respectively (Additional Table 4). In PEG(+) Mtarc2-KO mice fed a ND, 8 and 8 taxa that were overrepresented and underrepresented in females, and 5 and 6 taxa in males, respectively, and in PEG(+) C57BL/6 N mice, 7 and 13 taxa were overrepresented and underrepresented in females, and 10 and 13 taxa in males, respectively (Additional Table 4). Of the overrepresented genera, *Roseburia* was common to both PEG(-) and PEG(+) female groups, and *Roseburia*,* Romboutsia* and *Tuzzerella* were common to the male groups from both strains, while underrepresented *Paludicola* and *Parvibacter* and underrepresented *Paludicola* and *Corynebacterium* were common to all female and male groups of PEG(-) and PEG(+) mice, respectively (Table 5).

**Table 5:** List of differentially abundant genera distinguishing between T16 and T0 in normal diet (ND) and Western diet (WD)-fed mice that were not subjected to intestinal cleansing (polyethylene glycol [PEG(−)] groups) and those that underwent intestinal cleansing by oral gastric gavages with an aqueous solution of PEG [PEG(+) groups]

Females, Normal Diet							
Mtarc2-KO; PEG (-)	Mtarc2-KO; PEG(+)	C57BL/6 N; PEG (-)	C57BL/6 N; PEG (+)	Mtarc2-KO; PEG (-)	Mtarc2-KO; PEG (+)	C57BL/6 N; PEG (-)	C57BL/6 N; PEG (+)
Overrepresented taxa				Underrepresented taxa			
*Roseburia*	*Roseburia*	*Roseburia*	*Roseburia*	*Parvibacter*	*Parvibacter*	*Parvibacter*	*Parvibacter*
*Romboutsia*	*Romboutsia*	*Bifidobacterium*	*Bifidobacterium*	*Paludicola*	*Paludicola*	*Paludicola*	*Paludicola*
	*Clostridium sensu stricto 1*	*Intestinimonas*	*Intestinimonas*	*Dubosiella*	*Dubosiella*	*Bacteroides*	*Bacteroides*
	*Desulfovibrio*	*Muribaculum*	*Muribaculum*		*Helicobacter*	*Lachnospiraceae UCG-006*	*Lachnospiraceae UCG-006*
	*Oscillibacter*		*Alistipes*		*[Eubacterium] nodatum group*	*Lactococcus*	*Lactococcus*
	*Lachnoclostridium*		*A2*		*Corynebacterium*	*[Eubacterium] brachy group*	*[Eubacterium] brachy group*
	*Turicibacter*		*Clostridium sensu stricto 1*		*Enterorhabdus*	*Lactobacillus*	*Lactobacillus*
	*Lachnospiraceae NK4A136 group*				*[Eubacterium] fissicatena group*	*Helicobacter*	*Helicobacter*
						*Escherichia-Shigella*	*Escherichia-Shigella*
						*Corynebacterium*	*Corynebacterium*
							*Staphylococcus*
							*Dubosiella*
							*Enterorhabdus*

Males, Normal Diet							
*Mtarc2-KO; PEG (-)*	*Mtarc2-KO; PEG(+)*	*C57BL/6 N; PEG (-)*	C57BL/6 N; PEG (+)	Mtarc2-KO; PEG (-)	Mtarc2-KO; PEG (+)	C57BL/6 N; PEG (-)	C57BL/6 N; PEG (+)
Overrepresented taxa				Underrepresented taxa			
*Romboutsia*	*Romboutsia*	*Romboutsia*	*Romboutsia*	*Paludicola*	*Paludicola*	*Paludicola*	*Paludicola*
*Roseburia*	*Roseburia*	*Roseburia*	*Roseburia*	*Corynebacterium*	*Corynebacterium*	*Corynebacterium*	*Corynebacterium*
*Tuzzerella*	*Tuzzerella*	*Tuzzerella*	*Tuzzerella*	*[Eubacterium] fissicatena group*	*[Eubacterium] fissicatena group*	*Desulfovibrio*	*Desulfovibrio*
*Ileibacterium*	*Ileibacterium*	*Thermicanus*	*Thermicanus*	*Staphylococcus*	*Staphylococcus*	*Enterorhabdus*	*Enterorhabdus*
	*Methylobacterium-Methylorubrum*	*Intestinimonas*	*Intestinimonas*			*Helicobacter*	*Helicobacter*
		*[Eubacterium] oxidoreducens group*	*[Eubacterium] oxidoreducens group*		*Family XIII AD3011 group*	*Anaerotruncus*	*Anaerotruncus*
		*Oscillibacter*	*Oscillibacter*		*[Eubacterium] brachy group*	*Candidatus Saccharimonas*	*Candidatus Saccharimonas*
		*Alistipes*	*Alistipes*			*[Eubacterium] nodatum group*	*[Eubacterium] nodatum group*
			*A2*			*Parvibacter*	*Parvibacter*
			*Turicibacter*			*Erysipelatoclostridium*	*Erysipelatoclostridium*
						*Lachnospiraceae UCG-006*	*Lachnospiraceae UCG-006*
						*[Eubacterium] brachy group*	*[Eubacterium] brachy group*
							*Family XIII AD3011 group*

Females, Western Diet							
Mtarc2-KO; PEG (-)	Mtarc2-KO; PEG(+)	C57BL/6 N; PEG (-)	C57BL/6 N; PEG (+)	Mtarc2-KO; PEG (-)	Mtarc2-KO; PEG (+)	C57BL/6 N; PEG (-)	C57BL/6 N; PEG (+)
Overrepresented taxa				Underrepresented taxa			
*Clostridium sensu stricto 1*	*Clostridium sensu stricto 1*	*Mucispirillum*	*Mucispirillum*	*Paludicola*	*Paludicola*	*Paludicola*	*Paludicola*
*Romboutsia*	*Romboutsia*	*Lachnoclostridium*	*Lachnoclostridium*	*Candidatus Saccharimonas*	*Candidatus Saccharimonas*	*Candidatus Saccharimonas*	*Candidatus Saccharimonas*
*Lachnospiraceae NK4A136 group*	*Lachnospiraceae NK4A136 group*	*Alistipes*	*Alistipes*	*ASF356*	*ASF356*	*ASF356*	*ASF356*
	*Alistipes*	*Bacteroides*	*Bacteroides*	*Dubosiella*	*Dubosiella*	*Dubosiella*	*Dubosiella*
	*Lachnoclostridium*	*Blautia*	*Blautia*		*Parvibacter*	*Lachnospiraceae UCG-006*	
		*Intestinimonas*	*Intestinimonas*		*[Eubacterium] nodatum group*	*Helicobacter*	*Helicobacter*
		*Tuzzerella*	*Tuzzerella*			*[Eubacterium] brachy group*	*[Eubacterium] brachy group*
		*Bilophila*	*Bilophila*			*Lactobacillus*	*Lactobacillus*
		*Oscillibacter*	*Oscillibacter*			*Parvibacter*	*Parvibacter*
		*Parabacteroides*	*Parabacteroides*			*Anaerotruncus*	*Anaerotruncus*
		*Colidextribacter*	*Colidextribacter*			*Marvinbryantia*	*Marvinbryantia*
			*Muribaculum*			*[Eubacterium] fissicatena group*	*[Eubacterium] fissicatena group*
			*Anaerovorax*			*[Eubacterium] nodatum group*	*[Eubacterium] nodatum group*
						*Faecalibaculum*	*Faecalibaculum*
							*Lachnospiraceae UCG-006*

Males, Western Diet							
Mtarc2-KO; PEG (-)	Mtarc2-KO; PEG(+)	C57BL/6 N; PEG (-)	C57BL/6 N; PEG (+)	Mtarc2-KO; PEG (-)	Mtarc2-KO; PEG (+)	C57BL/6 N; PEG (-)	C57BL/6 N; PEG (+)
Overrepresented taxa				Underrepresented taxa			
*Romboutsia*	*Romboutsia*	*Romboutsia*	*Romboutsia*	*Paludicola*	*Paludicola*	*Paludicola*	*Paludicola*
*Turicibacter*	*Turicibacter*	*Roseburia*	*Roseburia*	*[Eubacterium] nodatum group*	*[Eubacterium] nodatum group*	*[Eubacterium] nodatum group*	*[Eubacterium] nodatum group*
*Tuzzerella*	*Tuzzerella*	*Tuzzerella*	*Tuzzerella*	*UBA1819*	*UBA1819*	*Parvibacter*	*Parvibacter*
*Roseburia*	*Roseburia*	*Intestinimonas*	*Intestinimonas*	*Lactobacillus*	*Lactobacillus*	*Lachnospiraceae UCG-006*	*Lachnospiraceae UCG-006*
*Blautia*	*Blautia*	*[Eubacterium] oxidoreducens group*	*[Eubacterium] oxidoreducens group*	*Anaerotruncus*	*Anaerotruncus*	*Candidatus Saccharimonas*	*Candidatus Saccharimonas*
*Lachnoclostridium*	*Lachnoclostridium*	*Oscillibacter*	*Oscillibacter*	*Muribaculum*	*Muribaculum*	*Desulfovibrio*	*Akkermansia*
*A2*	*A2*	*Alistipes*	*Bifidobacterium*	*Dubosiella*	*Dubosiella*	*Enterorhabdus*	*Dubosiella*
	*Enterorhabdus*	*Thermicanus*	*Blautia*	*Butyricicoccus*	*Butyricicoccus*	*Helicobacter*	*Parasutterella*
	*GCA-900,066,575*		*Bilophila*	*Marvinbryantia*	*Marvinbryantia*	*Anaerotruncus*	*Lactobacillus*
	*Bilophila*		*A2*		*Parabacteroides*	*Erysipelatoclostridium*	*Muribaculum*
	*Faecalibaculum*		*Colidextribacter*		*Corynebacterium*	*[Eubacterium] brachy group*	*Lachnospiraceae UCG-001*
			*Erysipelatoclostridium*			*Corynebacterium*	*[Eubacterium] fissicatena group*
			*Ileibacterium*				*Marvinbryantia*
			*Harryflintia*				*Lachnospiraceae NK4A136 group*

WD feeding of PEG(-) mice increased abundances of 3 and 11 taxa and decreased abundances of four and 14 taxa in Mtarc2-KO and C57BL/6 N females, and increased abundance of seven and eight taxa and decreased abundances of nine and 12 taxa in Mtarc2-KO and C57BL/6 N males, respectively (Table 5). In PEG(+) females fed a WD, five and 13 taxa were overrepresented and 6 and 14 taxa were underrepresented in Mtarc2-KO and C57BL/6 N groups, and 11 and 14 taxa were overrepresented and the other 11 and 14 taxa were underrepresented in male groups, respectively (Table 5). As shown in Table 5, three and 11 taxa overrepresented in Mtarc2-KO and C57BL/6 N females, four and 14 taxa underrepresented in Mtarc2-KO and C57BL/6 N females, seven and six taxa overrepresented in Mtarc2-KO and C57BL/6 N males, and nine and six taxa underrepresented in Mtarc2-KO and C57BL/6 N males were common to PEG(−) and PEG(+) groups.

Finally, the effects of FMT on taxa abundances were determined by comparing bacteria that differentiated NFMT mice and those with FMT from lean and obese human donors at the time point T16 of ND and WD feeding. Of 16 and 23 taxa that were overrepresented and underrepresented, respectively, in at least one of 16 FMT groups at the end of the experiment compared to corresponding NFMT groups, five overrepresented (*Butyricimonas*,* Odoribacter*,* Dubosiella*,* Escherichia-Shigella*,* Ileibacterium*) and three underrepresented (*Akkermansia*,* Alloprevotella*,* Ileibacterium*) taxa were found in at least one of mARC2-KO and C57BL/6 N B6 mice group (Table 6). *Butyricimonas* was not present in the gut microbiome of mice, but was detected in all donor samples, with abundance ranging from 0.53 to 0.89% in obese donors and from 1.21 to 1.33% in lean donors. Regardless of whether the stool samples were from lean or obese donors, FMT engrafted all groups of the mice with *Butyricimonas*, and increased abundances of *Odoribacter* was found in ND-fed C57BL/6 N females transplanted with feces from lean and obese, in ND-fed C57BL/6 N males transplanted with feces from lean donors, and in all but one mice groups fed a WD (Table 6). It should be noted that the abundance of *Alloprevotella*,* Akkermansia*,* Defluviitaleaceae UCG-011*,* Ileibacterium*,* Bifidobacterium* was found to be significantly higher in some FMT groups and lower in other groups than in their NFMT counterparts (Table 6).

**Table 6:** Unique and common bacteria between eight experimental groups of Mtarc2-KO and C57BL/6 N mice categorized by sex, genotype, and FMT donor phenotype (lean or obese)

Mtarc2-KO mice				C57BL/6 *N* mice			
Females		Males		Females		Males	
FMT(lean)	FMT(obese)	FMT(lean)	FMT(obese)	FMT(lean)	FMT(obese)	FMT(lean)	FMT(obese)
Overrepresented genera in mice on Normal Diet							
* Butyricimonas*	*Butyricimonas*	*Butyricimonas*	*Butyricimonas*	*Butyricimonas*	*Butyricimonas*	*Butyricimonas*	*Butyricimonas*
* Dubosiella*	*Alloprevotella*			*Odoribacter*	*Odoribacter*	*Odoribacter*	
	*Harryflintia*			*Akkermansia*		*Parasutterella*	
	*Defluviitaleaceae UCG-011*			*Ileibacterium*		*Bifidobacterium*	
Overrepresented genera in mice on Western Diet							
* Butyricimonas*	*Butyricimonas*	*Butyricimonas*	*Butyricimonas*	*Butyricimonas*	*Butyricimonas*	*Butyricimonas*	Butyricimonas
* Alloprevotella*	*Odoribacter*	*Odoribacter*	*Odoribacter*	*Odoribacter*	*Odoribacter*	*Odoribacter*	Odoribacter
* Escherichia-Shigella*	*Alloprevotella*	*Ileibacterium*	*Marvinbryantia*	*Escherichia-Shigella*	*Escherichia-Shigella*	*Bifidobacterium*	
* Paludicola*	*Roseburia*	*Paludicola*			*Dubosiella*		
	*Defluviitaleaceae UCG-011*	*Parvibacter*			*Faecalibaculum*		
Underrepresented genera in mice on Normal Diet							
* Lachnospiraceae UCG-008*	*Lachnospiraceae UCG-008*		*Alloprevotella*	*Ileibacterium*		*Alloprevotella*	*ASF356*
* Intestinimonas*			*Mucispirillum*	*Bifidobacterium*		*Parabacteroides*	*Muribaculum*
* Anaerotruncus*				*Faecalibaculum*			
* Blautia*				*Butyricicoccus*			
Underrepresented genera in mice on Western Diet							
* Defluviitaleaceae UCG-011*		*Akkermansia*	*Ileibacterium*	*Lachnospiraceae UCG-008*	*Candidatus Saccharimonas*	*Akkermansia*	*Akkermansia*
* Lachnoclostridium*			*Colidextribacter*	*[Eubacterium] fissicatena group*		*Muribaculum*	*Muribaculum*
* GCA-900066575*				*Enterorhabdus*		*Marvinbryantia*	*Marvinbryantia*
						*GCA-900066575*	*Alloprevotella*
						*Butyricicoccus*	*Candidatus Saccharimonas*

**Fig. 7:**
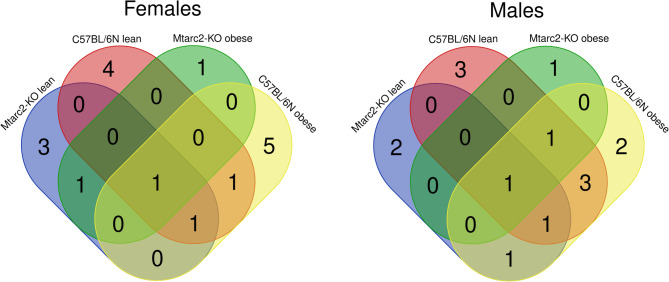
Numbers of genera distinguishing between not transplanted (NFMT) mice groups and those transplanted with fecal extracts from lean human donors [FMT (lean)] and obese [FMT (obese)] at the time point T16 of WD feeding

Separate Venn diagrams were generated for females and males (Fig. 7). In females, four (*A2*, *Enterorhabdus*, *Odoribacter*, and *Defluviitaleaceae UCG-011*) and three (*Bifidobacterium*, *Ileibacterium*, and *Paludicola*) genera were unique to C57BL/6 N and Mtarc2-KO mice with FMT from lean donors, respectively. By contrast, five (*Candidatus Saccharimonas*, *Parabacteroides*, *Dubosiella*, *Helicobacter*, and *Eubacterium oxidoreducens*) and one (*Roseburia*) were unique to C57BL/6 N and Mtarc2-KO mice with FMT from obese donors, respectively (Fig. 7). In males, three (*Bifidobacterium*, *Enterorhabdus*, and *Lachnospiraceae UCG-008*) and two (*Ileibacterium* and *Paludicola*) genera were unique to C57BL/6 N and Mtarc2-KO mice with FMT from lean donors. In mice with FMT from obese donors, we observed two genera (*Muribaculum* and *Blautia*) and one genus (*ASF356*) unique to C57BL/6 N and Mtarc2-KO mice, respectively. One genus (*Alloprevotella*) was shared between C57BL/6 N and Mtarc2-KO with FMT from obese and lean donors, while three genera (*GCA-900066575*, *Akkermansia*, and *Odoribacter*) were shared between C57BL/6 N mice with FMT from lean and obese donors. *Butyricimonas* was the only genus present in all FMT groups.

**Fig. 8:**
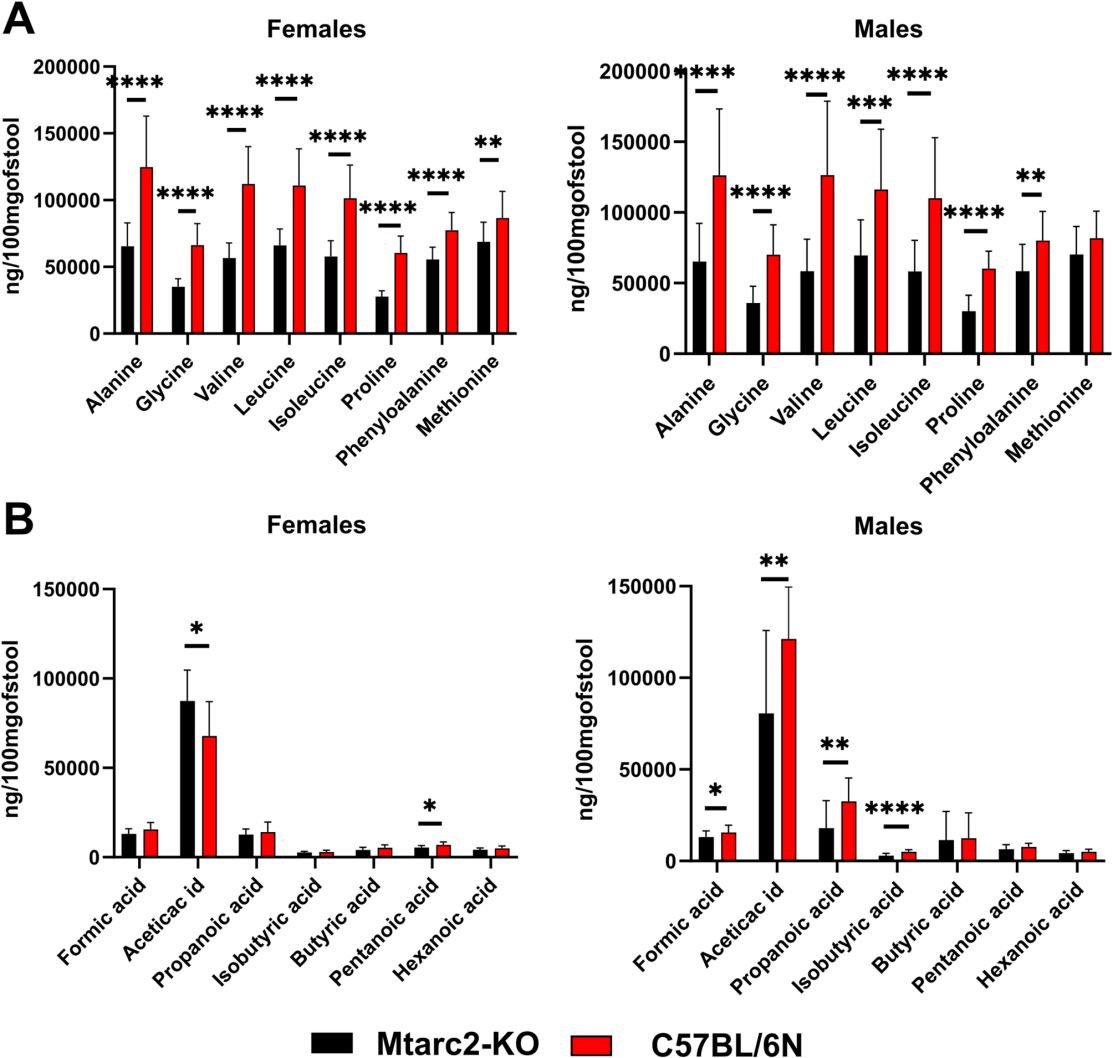
Relative abundance of amino acids (**A**) and short chain fatty acids (**B**) in PEG(-) Mtarc2-KO and C57BL/6 N mice at the beginning (T0) of experiment. Statistical significance: * *p* < 0.05; ** *p* < 0.01; *** *p* < 0.001; **** *p* < 0.0001

GC-MS-based analyses identified eight SCFAs (formic acid, acetic acid, propanoic acid, isobutyric acid, butanoic acid, pentanoic acid, and hexanoic acid) and eight AAs (alanine, glycine, valine, leucine, isoleucine, proline, phenylalanine, and methionine) in fecal sample extracts. Similar to the significant differences in bacterial abundances between Mtarc2-KO and C57BL/6 N groups at the beginning of the experiment, the relative abundances of all eight AAs studied were significantly higher in C57BL/6 N females than in Mtarc2-KO females and for all but one AA in males (Fig. 8). However. In comparison, the abundances of formic, acetic, propanoic, and isobutyric acids were significantly higher in C57BL/6 N males than in Mtarc2-KO males, and the abundance of acetic acid was significantly higher. The abundance of pentanoic acid was significantly lower in Mtarc2-KO females than in C57BL/6 N females (Fig. 8).

**Fig. 9:**
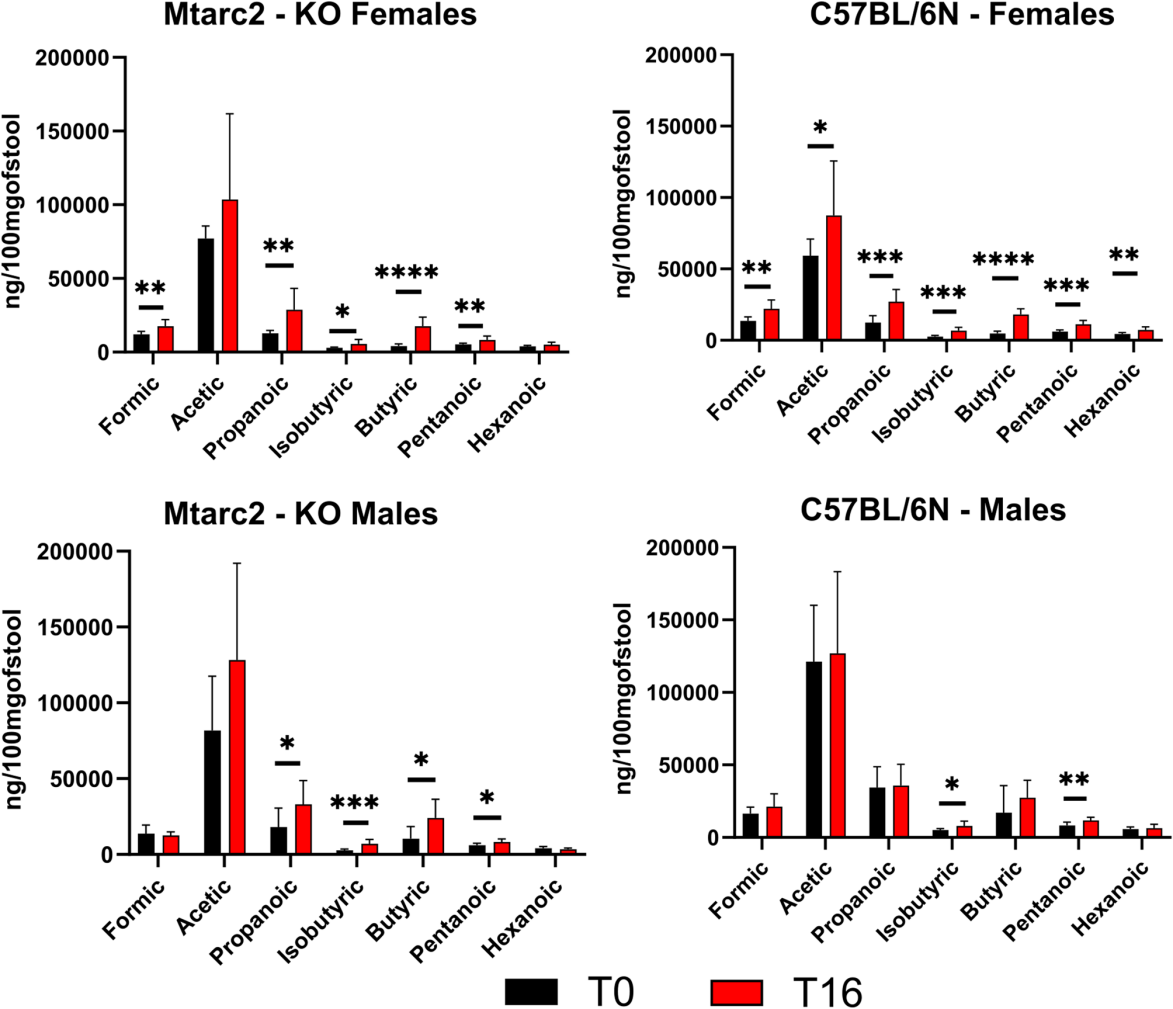
Comparison of relative abundance of short chain fatty acids (SCFAs) between T16 and T0 in mice fed WD. Statistical significance: * *p* < 0.05; ** *p* < 0.01; *** *p* < 0.001; **** *p* < 0.0001

Pairwise comparisons of metabolite abundances between T16 and T0 revealed that in Mtarc2-KO mice fed a WD, the relative abundances of phenylalanine and methionine were significantly higher at T16 than at T0 in females, while AA abundances did not change in males. In WD-fed C57BL/6 N mice, there were significantly lower abundances of alanine and proline, and significantly lower abundances of all AAs except phenylalanine and methionine, at T16 than at T0 in females and males, respectively (Additional Fig. 15). Of the seven SCFAs tested, five (formic, propanoic, isobutyric, butanoic, and pentanoic acids), four (propanoic, isobutyric, butanoic, and pentanoic acid), all seven, and two (isobutyric and pentanoic acids) were significantly higher at T16 than at T0 in Mtarc2-KO females and males and C57BL/6 N females and males, respectively, fed a WD (Fig. 9).

**Fig. 10:**
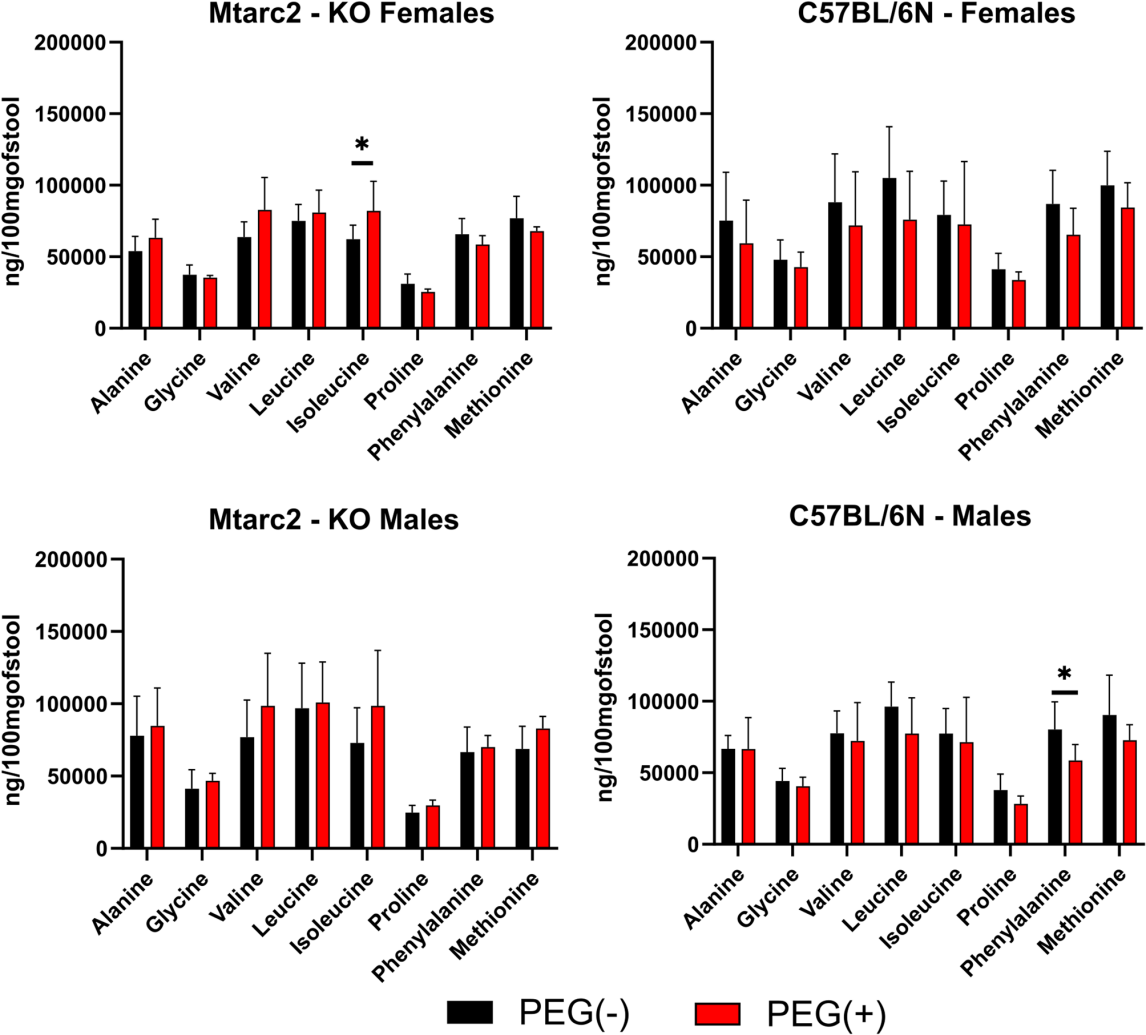
Comparison of relative abundance of amino acids (A.As) between PEG(-) and PEG(+) WD fed groups at the end of the experiment. Statistical significance: * *p* < 0.05

Compared with PEG(−) groups, PEG administration did not modify AAs profiles in WD-fed groups at the end of the experiment; however, it did result in significantly higher abundances of formic acid in Mtarc2-KO females and males and C57BL/6 N females, and significantly lower abundances of acetic, propanoic, and butyric acids, regardless of strain or sex (Fig. 10 and Additional Fig. 16).

Finally, we compared the metabolite abundance between WD-fed NFMT mice and mice with FMT from lean or obese human donors. As shown in Additional Fig. 17, in fecal samples collected at the end of the experiment, WD-fed Mtarc2-KO females with FMT from lean donors had significantly higher abundances of glycine, proline, phenylalanine, and methionine compared with NFMT mice; no differences were found in any other mice with FMT from either lean or obese donors. Compared with NFMT, FMTs from lean donors resulted in a significantly higher abundance of butyric acid in Mtarc2-KO females and a significantly lower abundance of formic, pentanoic, and hexanoic acids in Mtarc2-KO males; FMTs from obese donors resulted in a significantly lower abundance of butyric and pentanoic acids in Mtarc2-KO males (Additional Fig. 18). No AAs or SCFAs abundances were affected by FMT among C57BL/6 N mice (Additional Figs. 17 and 18).

## Discussion

Diet quality and genetic background are critical factors that influence gut microbiota compositions in humans and animals [1]. In this study, we analyzed the impact of high-fat (WD) feeding on phenotypic outcomes, liver accumulation of FAs, and modifications of the gut microbial community, as well as SCFAs and AAs abundance, in Mtarc2-KO mice and C57BL/6 N background mice.

We first compared differences observed in mice that were conventionally raised, i.e., those not subjected to a reduction of the intestinal microbiota load by PEG administration. C57BL/6 N and Mtarc2-KO males, but not females on the WD, gained more body weight than those on the ND. The increase in body weight of Mtarc2-KO males and females fed a WD for 16 weeks was significantly lower than that observed in the corresponding groups of C57BL/6 N mice, and Mtarc2-KO females fed a WD had lower visceral and gonadal fat than ND-fed mice. Although fatty liver was found in all mice on the WD, it was highest in C57BL/6 N males and lowest in Mtarc2-KO females. The background pattern of liver FAs accumulation was similar in both mouse strains independent of sex: WD feeding resulted in increased levels of lauric acid, myristic acid, trans-vaccenic acid, α-linolenic acid, eicosapentaenoic acid, and docosahexaenoic acid and decreased and arachidonic acid and adrenic acid, which occurred to the same extent in all mice. WD feeding also increased serum levels of total cholesterol and HDL-C in all mice, but increased serum triglyceride levels only in C57BL/6 N mice.

Background α-diversity at the beginning of the experiment, as analyzed by the Shannon and Chao1 indexes, did not differentiate females from males within strains and did not differentiate between strains. By contrast, β-diversity of the bacterial structure differentiated females from males within strains and differentiated between strains; this was in line with 20 and 21 genera having differential abundances between Mtarc2-KO and C57BL/6 N mice in female and male groups, respectively. Of these, *Bifidobacterium*,* Ileibacterium*,* [Eubacterium] xylanophilum group*,* Coriobacteriaceae UCG-002*, and *Harryflintia* were overrepresented and *Paludicola*,* Lachnospiraceae UCG-006*,* Lachnospiraceae NK4A136 group*,* Mucispirillum*,* Butyricicoccus*,* Lactococcus*, and Defluviitaleaceae UCG-011 were underrepresented in both sexes of Mtarc2-KO mice compared with C57BL/6 N mice.

WD feeding significantly affected α-diversity in C57BL/6 N mice and Mtarc2-KO females compared with ND-fed mice, as well as β-diversity in all groups at T16 compared with T0. Of 58 unique taxa whose abundance differed in mice on the WD at T16 compared with T0, seven and 16 genera differed in the female and male groups of Mtarc2-KO mice, respectively, and 25 and 20 differed in the female and male groups of C57BL/6 N mice, respectively. Of these, the overrepresented *Romboutsia*,* Roseburia*, and *Tuzzerella* were common to both strain male groups, the underrepresented *Paludicola*,* Candidatus Saccharimonas*,* ASF356 and Dubosiella* were common to both strain female groups, and the underrepresented *Paludicola*, [Eubacterium] nodatun group, and *Anaerotruncus* were common to both strain male groups.

The metabolic function of the gut microbiota results mainly from metabolites generated by the intestinal microbiota, of which SCFAs are the largest group. Straight-chain SCFAs (acetate, propionate, and butyrate) are produced by the fermentation of dietary fiber and resistant starch in the intestinal lumen [34]. Butyrate is produced mainly by *Clostridium*, *Eubacterium*, and *Fusobacterium* genera, of which the most productive are *Clostridium leptum*, *Roseburia* spp., *Faecalibacterium prausnitzii*, and *Coprococcus* spp. Acetate is produced mainly by *Bifidobacteria* spp., while propionate is a metabolite of *Bacteroidetes* and *Propionibacterium* [35]. Branched-chain SCFAs, including isobutyric acid, are made from the branched-chain AAs leucine, isoleucine, and valine. These compounds are absorbed into the systemic circulation by passive diffusion and active transport, where they influence the regulation of appetite by binding with G-protein-coupled free fatty acid receptor 3 and stimulate leptin secretion by adipose tissue, thus taking part in the maintenance of energy homeostasis [36]. Bacterial metabolic processes in distal parts of the colon may be related to the availability of AAs [37].

In this study, the background abundances of all eight (females) and seven (males) AAs tested at T0 were significantly higher in C57BL/6 N mice than in Mtarc2-KO mice. The abundance of formic, acetic, propanoic, and isobutyric acid was higher, while the abundance of acetic acid was lower, in Mtarc2-KO females and males, respectively, than in C57BL/6 N mice. WD feeding resulted in increased abundances of two (females) and zero (males) AAs in Mtarc2-KO mice and decreased the abundances of two (females) and six (males) AAs in C57BL/6 N mice. Of the seven SCFAs tested, five, four, seven, and three were higher at T16 than T0 in Mtarc2-KO females and males and C57BL/6 N females and males fed a WD, respectively. Of these, the abundance of isobutyric, butyric, and propanoic acids was increased at T16 in all mice. Although metabolite abundances, especially for AAs, clearly differentiated the mouse strains at the beginning of the experiment, WD-related differences in AAs abundance were relatively weak compared with the substantial differences in SCFAs abundance detected at the end of the experiment, independent of the strain or sex of mice. These results suggest that a WD increases the production of SCFAs regardless of body weight gain.

In general, we observed significant differences in phenotypic, metagenomic, and metabolomic measures between conventionally raised mouse strains and sex. The Mtarc enzyme complex, together with Cyb5b and Cyb5R, is located on the outer mitochondrial membrane and functions as a reducer of nitrogen-containing functional groups [38–40]. In humans, the highest levels of Mtarc2 mRNA are in the kidney, thyroid, liver, and small intestine [41]. Mtarc proteins are also present in peroxisomal membranes without Cyb5b and Cyb5R, which may indicate a function of Mtarc proteins other than nitrogen reduction. Considering the general function of peroxisomes, Mtarc may be involved in the regulation of lipid metabolism [19]. In fact, our yet unpublished study found 232 upregulated and 352 downregulated liver transcripts in Mtarc2-KO males compared to wild-type male mice fed a high-fat diet. The REACTOME database-based functional analyses among downregulated mRNAs revealed a significant overrepresentation of transcripts encoding proteins involved in *Metabolism of lipids* (R-MMU-556833), *Peroxisomal lipid metabolism* (R-MMU-390918) and *alpha-linolenic (omega3) and linoleic (omega6) acid metabolism* (R-MMU-2046104). However, although we confirmed our previous findings, which showed significantly lower body weight gain and total fat in Mtarc2-KO mice than in wild-type mice [25], liver accumulation of FAs and steatosis grade depended much less on the strain or sex of mice than other measures. Therefore, a precise molecular mechanism underlying a potential connection between lipid metabolism and the Mtarc2 enzyme remains unclear.

Human microbiota-associated (HMA) mice allow the study of the relationship between the intestinal microbiota and diseases, including diet-induced obesity and non-alcoholic fatty liver disease [42–44]. HMA models refer to germ-free mice transplanted with human fecal samples [33]. As an alternative to classical HMA mice, conventional (i.e. not germ-free) mice are transplanted with fecal microbiota after prior antibiotic pre-treatment or bowel cleansing [45, 46]. Subsequently, we have engrafted the alimentary tract of mice with human stool using the method established by Wrzosek et al., which allows the detection of human bacteria after four weeks, even if only one FMT was performed [45]. Although it is not clear how many FMT dosages are essential to sustain the donor microbiota in the recipient in the long-term, in this study we administered fecal suspensions at weekly intervals during the whole 16-week protocol.

Unexpectedly, the cleansing procedure itself resulted in significantly lower body weight gain in males fed ND and in both females and males fed WD among C57BL/6 N mice compared with PEG(−) mice, but did not change the body weight of the Mtarc2-KO mice. Compared with a water-only gavage, PEG administration significantly increased liver weight in ND-fed C57BL/6 N males and WD-fed Mtarc2-KO females. Independent of mouse strain, sex, or diet, intestinal cleansing increased liver accumulation of trans-vaccenic acid, erucic acid, nervonic acid, and α-linolenic acid and decreased accumulation of adrenic acid, di-homo-gamma-linolenic acid, and arachidonic acid. However, it did not affect the microscopic findings of fatty liver.

In WD-fed mice, intestinal cleansing decreased the Shannon index in females and increased the Chao1 index in female and male C57BL/6 N mice, while in Mtarc2-KO mice, it increased the Chao1 index only in males. Compared with PEG(−) groups, PEG(+) groups had fewer and different differentiating bacteria between T16 and T0, including *Lachnospiraceae NK4A136 Dubosiella* and *Paludicola* in Mtarc2-KO females, *Romboutsia*,* Dubosiella*,* Paludicola*, and *UBA1819* in Mtarc2-KO males, *Dubosiella*,* Marvinbryantia*, and *Faecalibaculum* in C57BL/6 N females, and *Blautia*,* Bilophila*,* Dubosiella* and *Defluviitaleaceae UCG-011* in C57BL/6 N males. While PEG administration did not modify profiles of AAs in WD-fed mice at the end of the experiment, it significantly increased the abundance of formic acid in WD-fed Mtarc2-KO mice and C57BL/6 N females and decreased the abundance of acetic, propanoic, and butyric acids, regardless of strain or sex.

While no effect of FMT on body weight was observed in C57BL/6 N mice, it decreased weight gain in Mtarc2-KO females fed an ND and males fed a WD. FMT from either lean or obese donors increased liver weight in WD-fed Mtarc2-KO females and gonadal fat weight in ND-fed Mtarc2-KO females. FMT from lean donors decreased liver weight in WD-fed Mtarc2-KO males. FMT did not affect liver microscopic findings, but some modulatory effect of transplantation with the stool of lean or obese donors on liver FA accumulation was observed, particularly in C57BL/6 N mice. Under WD conditions, FMT from lean humans increased both α-diversity indexes in C57BL/6 N males, while FMT from obese humans increased the Shannon index in C57BL/6 N females.

The Euclidean metric based on taxa abundance quantified differences in the intestinal microbiota between FMT and NFMT groups, which was further confirmed by the specific taxa analysis. However, *Butyricimonas* was the only genus present in all FMT groups and could be considered a marker of successful transplantation of human microbiota into mice. The other changes in taxa abundances were rather group-specific. Of these, the increased abundance of *Odoribacter* was found in ND-fed C57BL/6 N females transplanted with feces from lean and obese, in ND-fed C57BL/6 N males transplanted with feces from lean donors, and in all eight but one mice groups fed a WD. Additionally, both the fold change and the statistical significance of the differences were much higher for *Butyricimonas* than for *Odoribacter*. Through multivariable Mendelian randomization analysis Liu et al. [47] demonstrated that the *Butyricimonas* genus plays a direct role in elevating the risk of medication-induced obesity, highlighting a causal link between gut microbiota and distinct obesity subtypes. *Butyricimonas virosa* was tested in a mouse model of WD-induced obesity to explore its potential metabolic benefits [48]. Both live and heat-killed *B. virosa* improved body weight, serum glucose levels, insulin resistance, and liver steatosis in WD-fed mice. The treatment activated the GLP-1 receptor and PPARα in the liver and upregulated insulin receptor substrates 1 and 2, toll-like receptor 5, and zonula occludens 1 expression in the ileum. Notably, the glucose-regulating effects of *B. virosa* were linked to GLP-1 receptor activation in the liver rather than gut colonization or butyrate production by the bacteria.

Our analyses of the gut bacterial community structure and taxonomy identified only a few reproducible modifications related to the 16-week FMT procedure. Although FMT did not affect metabolite abundances in any of the WD-fed C57BL/6 N mice group, in Mtarc2-KO mice, FMT from lean donors increased the abundance of four AAs and butyric acid in females and decreased the abundance of formic, pentanoic, and hexanoic acids in males. The microbiota from obese donors decreased the abundance of butyric and pentanoic acids in Mtarc2-KO males. However, FMT did not impact fatty liver grade, as opposed to the effect of the PEG treatment itself.

As reported previously, gut microbiota manipulation or supplementation may restore a community associated with a healthy condition, enabling research on causal links between gut dysbiosis and negative outcomes [43]. FMT of a wild boar, which has leaner muscle and less fat than a domestic pig, prevented WD-induced obesity and altered lipid metabolism in a mouse model of obesity [49]. *Lactobacillus acidophilus* reversed WD-induced gut dysbiosis, and its anti-obesity effect was transmissible via horizontal feces transfer from *L. acidophilus*-treated mice to WD-fed mice [50]. The transplantation of healthy intestinal flora successfully reversed gut microbiota dysbiosis, particularly the decline of *Akkermansia*, in obese mice [49]. Horizontal FMT from ND-fed mice to WD-fed mice conferred anti-obesity effects, possibly by modulating gut microbiota composition [10]. By contrast, no significant impact on the body weight of C57BL/6NCrSIc mice was found after FMT from *Suncus murinus*, an obesity-resistant animal [7].

Another study using FMT from obese twin donors to germ-free (GF) mice fed ND or WD increased total body and fat mass, as well as obesity-associated metabolic phenotypes [8], and gut microbiota transferred from a genetically obese human to GF mice promoted the onset of liver steatosis by impacting hepatic lipid metabolism [51]. Increased epididymal fat weight and aggravated hepatic steatosis and inflammation were reported in GF mice inoculated with feces from patients with nonalcoholic steatohepatitis (NASH) and fed a HFD [52]. A transfer of fecal microbial communities from donors with hepatic steatosis grade 3 to recipient mice, after an antibiotic treatment and wash-out period, resulted in mouse liver lipid accumulation [53]. The other study conducted in mice pretreated with a mixture of antibiotics and colonized with microbiota originating from a patient with NAFLD or a healthy lean individual and fed a high-fructose, high-fat diet (2HFD) allowed the development of an early-phase NAFLD model in mice. The mice were overweight and had more adipose tissues and liver steatosis compared to the healthy microbiota recipient mice [54]. The ecological differences between the NAFLD patient and healthy individual were driven by some genera, including *Bacteroides*,* Alistipes*,* Parabacteroides*,* Desulfovibrio* and *Bilophila* [54]. Therefore, transplanting the human microbiota to mice may be a factor contributing to the early phases of NAFLD. However, while autologous FMT, collected during the weight-loss phase and administrated in the regain phase in obese patients, could preserve the weight loss and glycemic control associated with specific microbiome signatures [55], a randomized, placebo-controlled pilot study of the effects of FMT derived from a lean donor did not reduce BMI in obese, metabolically uncompromised patients [56]. Thus, whether FMT from obese donors can promote an obese phenotype, or whether FMT from lean donors can reverse an obese phenotype, remains unresolved. In contrast to previous studies, our study demonstrated that FMT did not transfer a human obese phenotype to either conventional C57BL/6 N mice, or weight gain-resistant Mtarc2-KO mice, although some modifications of intestinal microbiota and metabolite profiles were noted.

These discrepancies could result from several limitations of our study. Firstly, the FMT protocol was repeated according to the method previously established by other authors [45] without further evaluation of the optimal frequency and duration of the transplantation. Secondly, although the PEG intestinal cleansing procedure itself affected the gut bacterial structure more than the transplantation of human microbiota into recipient mice, we did not compare the bacteria-reduction strategy with alternatives, such as antibiotic treatment. Thirdly, although we confirmed the distinct microbiota structure of lean and obese stool donors in this study, we did not assess how to optimize donor selection. Finally, a relatively small sample size of mice in groups with a large number of groups studied could affect the power of statistical testing. All these limitations might have precluded the detection of differences between groups.

In conclusion, although several clinical trials of the application of FMT for a range of disorders, including obesity, insulin resistance, and metabolic syndrome, are being conducted, our study raises new questions rather than resolving those previously posed. Therefore, we agree with the recently stated view [33] that, because of the enormous complexity of relationships between the gut microbiome and host, it is nearly impossible to predict the results of FMT or determine the function of a specific microbe in the development of obesity.

## Supplementary Information

Supplementary Material 1: Fig. 1. Microbiota analysis of FMT donors classified as normal or obese. (A) Alpha diversity (Shannon index) showing reduced microbial diversity in donors with obesity compared to normal-weight donors. (B) Principal Component Analysis (PCA) illustrating separation of samples based on microbial community composition. (C) Relative abundance of dominant bacterial families in the microbiota also separates normal from obese donors.

Supplementary Material 2: Fig. 2. Effects of PEG treatment in Mtarc2-KO (A) and C57BL/6N mice (B) and fecal microbiota transplant (FMT) in Mtarc2-KO (C) and C57BL/6N mice (D) fed ND and WD at 16 weeks of body weight gain compared to control PEG(-) mice and PEG(+) mice that did not undergo FMT, respectively. Values are expressed as mean ± SD, *n* = 5–6 mice per group. * *p* < 0.05, *** *p* < 0.001.

Supplementary Material 3: Fig. 3. Comparisons of liver weights (A), visceral fat weights (B), gonadal fat weights (C), serum total cholesterol concentrations (D), low-density lipoprotein cholesterol (HDL) concentrations (E) and triglyceride concentrations (F) between groups of Mtarc2-KO and C57BL/6N mice that were fed a normal diet (ND) and those fed a Western Diet (WD) for 16 weeks. Values are expressed as mean ± SD, *n* = 8–10 mice per group. * *p* < 0.05, ** *p* < 0.01, *** *p* < 0.001 and **** *p* < 0.0001.

Supplementary Material 4: Fig. 4. Comparisons of liver weights (A), visceral fat weights (B), and gonadal fat weights (C) between groups of mice that were not treated [PEG (-)] and were treated with PEG [PEG (+)] at 4 monthly intervals. Values are expressed as mean ± SD, *n* = 8–10 mice per group. * *p* < 0.05.

Supplementary Material 5: Fig. 5. Comparisons of serum concentrations of total cholesterol (A), low-density lipoprotein cholesterol (HDL) (B), and triglyceride (C) between mice groups that were not treated [PEG (-)] and were treated with PEG [PEG (+)] at 4 monthly intervals. Values are expressed as mean ± SD, *n* = 8–10 mice per group. * *p* < 0.05, ** *p* < 0.01.

Supplementary Material 6: Fig. 6. Comparisons of liver, visceral fat and gonadal fat weights of Mtarc2-KO (A,C,E) and C57BL/6N (B,D,F) mice fed ND or WD that were only PEG treated [PEG/NFMT] and those transplanted with fecal microbiota isolated from lean [PEG/FMT(lean)] of obese [PEG/FMT(obese)] human donors. Values are expressed as mean ± SD, *n* = 8–10 mice per group. * *p* < 0.05, ** *p* < 0.01.

Supplementary Material 7: Fig. 7. Comparisons of serum concentration of total cholesterol, HDL and triglycerides in Mtarc2-KO (A,C,E) and C57BL/6N (B,D,F) mice fed ND or WD that were only PEG treated [PEG/FT(-)] and those transplanted with fecal microbiota isolated from lean (PEG/LFT) of obese (PEG/OFT) human donors. Values are expressed as mean ± SD, *n* = 8–10 mice per group. * *p* < 0.05, ** *p* < 0.01.

Supplementary Material 8: Fig. 8. Histopathology examination showing normal (A) and fatty liver (B).

Supplementary Material 9: Fig. 9. Comparisons of liver fatty acid concentrations in Mtarc2-KO and C57BL/6N mice fed ND or WD that were only PEG treated [PEG/NFMT] and those transplanted with fecal microbiota isolated from lean [PEG/FMT(lean)] of obese [PEG/FMT(obese)] human donors. Values are expressed as mean ± SD, *n* = 8–10 mice per group. * *p* < 0.05, ** *p* < 0.01.

Supplementary Material 10: Fig. 10. ∝-diversity analyzed by the Shannon and Chao1 indexes in fecal samples collected at the beginning (T0) of experiment.

Supplementary Material 11: Fig. 11. ∝-diversity analyzed by the Shannon and Chao1 indexes in fecal samples collected at the beginning (T0) and the end (T16) of experiments from Mtarc2-KO and C57BL/6N mice fed WD mice fed a WD. Statistical significance: * *p* < 0.05; ** *p* < 0.01.

Supplementary Material 12: Fig. 12. Principal coordinate analysis (PCoA) using the Euclidean metric of fecal samples collected at the beginning (T0) and the end (T16) of experiments from Western Diet (WD)-fed mice. Each dot represents a single sample.

Supplementary Material 13: Fig. 13. ∝-diversity analyzed by the Shannon and Chao1 indexes in fecal samples collected at T16 from PEG(-) and PEG(+) groups of mice fed a WD. Statistical significance: * *p* < 0.05; ** *p* < 0.01.

Supplementary Material 14: Fig. 14. ∝-diversity analyzed by the Shannon and Chao1 indexes in fecal samples collected at T16 from WD-fed mice not transplanted (NFMT) and transplanted with fecal extracts of lean [FMT(lean)] and obese [FMT (obese)] human donors. Statistical significance: * *p* < 0.05.

Supplementary Material 15: Fig. 15. Comparison of relative abundance of amino acids between T16 and T0 in mice fed WD. Statistical significance: * *p* < 0.05; ** *p* < 0.01; *** *p* < 0.001; **** *p* < 0.0001.

Supplementary Material 16: Fig. 16. Comparison of relative abundance of short chain fatty acids (SCFAs) between PEG(-) and PEG(+) WD fed groups at the end of the experiment. Statistical significance: * *p* < 0.05; ** *p* < 0.01; **** *p* < 0.0001.

Supplementary Material 17: Fig. 17. Comparison of relative abundance of amino acids (A.As) tested at the end of experiment between WD-fed mice which were not transplanted (NFMT) and those transplanted with fecal extracts of lean [FMT(lean)] and obese [FMT(obese)] human donors. Statistical significance: * *p* < 0.05; ** *p* < 0.01; *** *p* < 0.001.

Supplementary Material 18: Fig. 18. Comparison of relative abundance of short chain fatty acids (SCFAs) tested at the end of experiment between WD-fed mice which were not transplanted (NFMT) and those transplanted with fecal extracts of lean [FMT(lean)] and obese [FMT(obese)] human donors. Statistical significance: * *p* < 0.05.

Supplementary Material 19: Table 1. Effects of high fat diet (WD) feeding for 16 weeks on individual hepatic fatty acid concentrations (µg/g of sample) in Mtarc2-KO and C57BL/6N females and males.

Supplementary Material 20: Table 2. Effects of intestinal cleansing by PEG treatment on individual liver fatty acid concentrations (µg/gm of sample) in mARC2-KO and C57BL6/W females and males.

Supplementary Material 21: Table 3. Taxonomic analysis of fecal samples collected at the beginning of experiments (time point T0) in Mtarc2-KO females and males compared with C57BL/6N mice.

Supplementary Material 22: Table 4. Tax differentiated between T16 and T0 in at least one PEG(−) groups.

## Data Availability

The datasets presented in this study can be found in online repositories. The names of the repository and accession number can be found below: https://www.ncbi.nlm.nih.gov/bioproject/1224054.
