# Supplementary figures and images for: Western Diet and fecal microbiota transplantation alter phenotypic, liver fatty acids, and gut metagenomics and metabolomics in Mtarc2 knockout mice
Source: Genes Nutr. 2025 May 29;20:13. doi: 10.1186/s12263-025-00772-x (PMC12121045; doi:10.1186/s12263-025-00772-x)

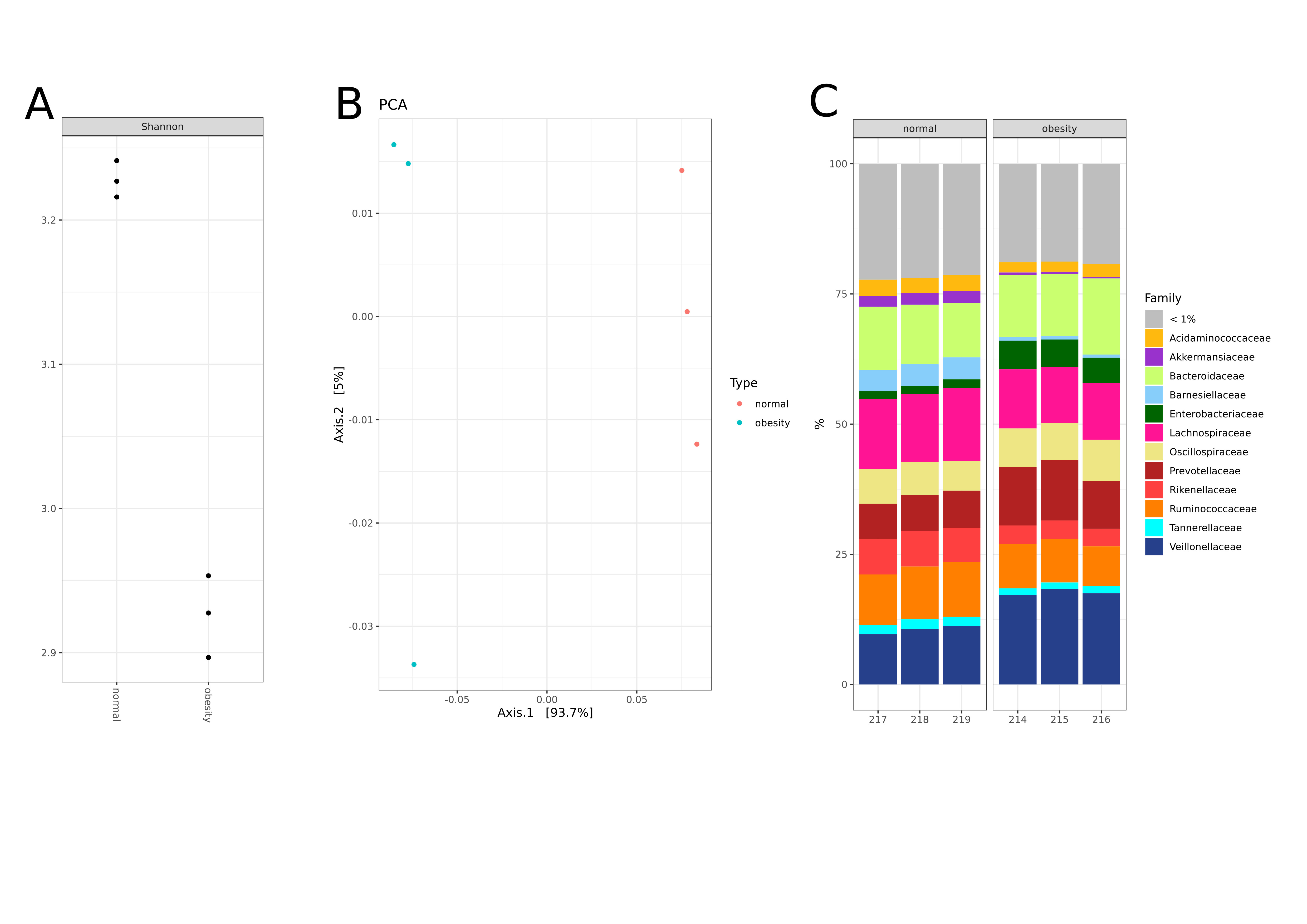

Supplement: Supplementary file 1 — Supplementary Material 1: Fig. 1. Microbiota analysis of FMT donors classified as normal or obese. (A) Alpha diversity (Shannon index) showing reduced microbial diversity in donors with obesity compared to normal-weight donors. (B) Principal Component Analysis (PCA) illustrating separation of samples based on microbial community composition. (C) Relative abundance of dominant bacterial families in the microbiota also separates normal from obese donors. [file 12263_2025_772_MOESM1_ESM.jpg]

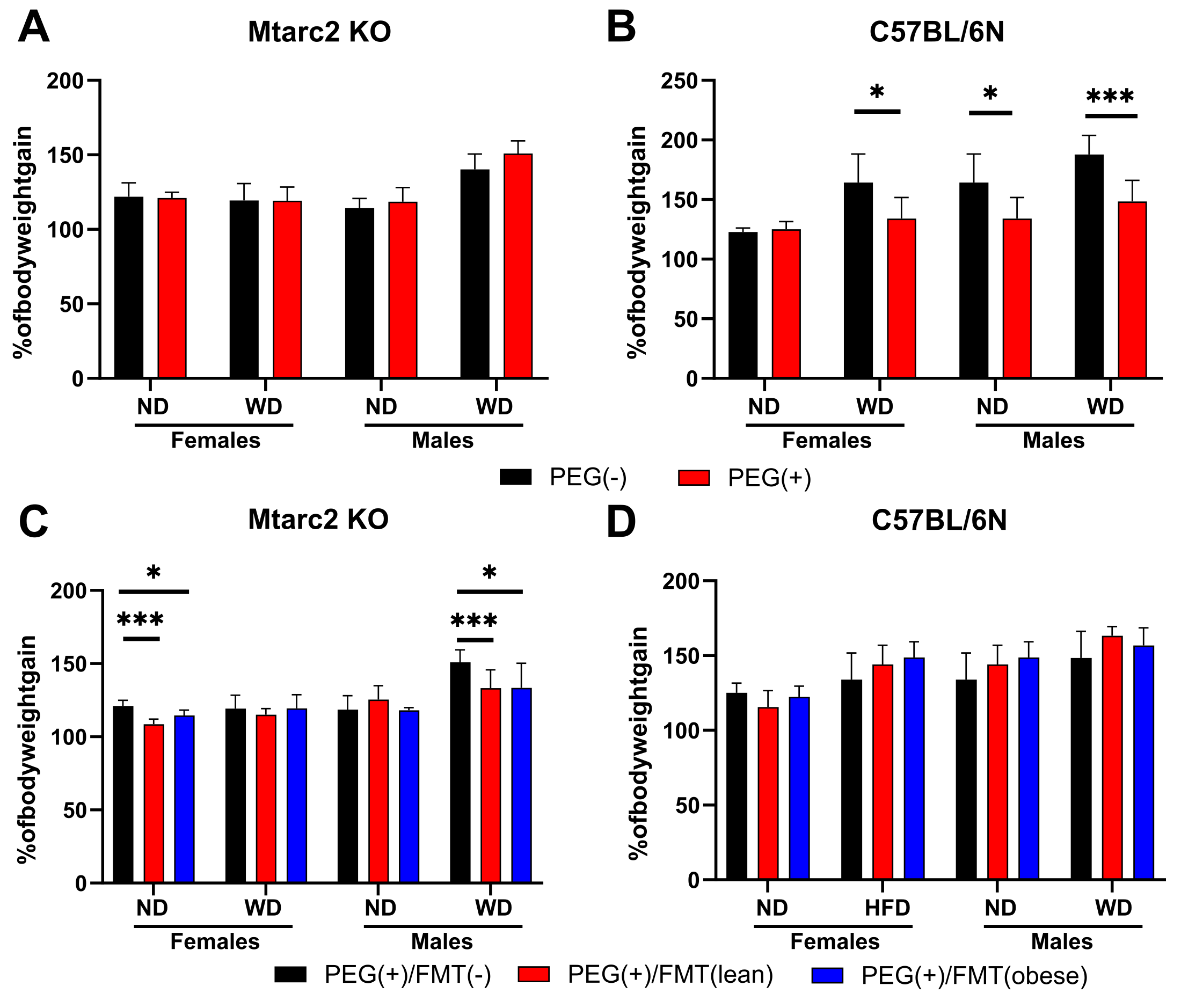

Supplement: Supplementary file 2 — Supplementary Material 2: Fig. 2. Effects of PEG treatment in Mtarc2-KO (A) and C57BL/6N mice (B) and fecal microbiota transplant (FMT) in Mtarc2-KO (C) and C57BL/6N mice (D) fed ND and WD at 16 weeks of body weight gain compared to control PEG(-) mice and PEG(+) mice that did not undergo FMT, respectively. Values are expressed as mean ± SD, n = 5–6 mice per group. * p < 0.05, *** p < 0.001. [file 12263_2025_772_MOESM2_ESM.tiff]

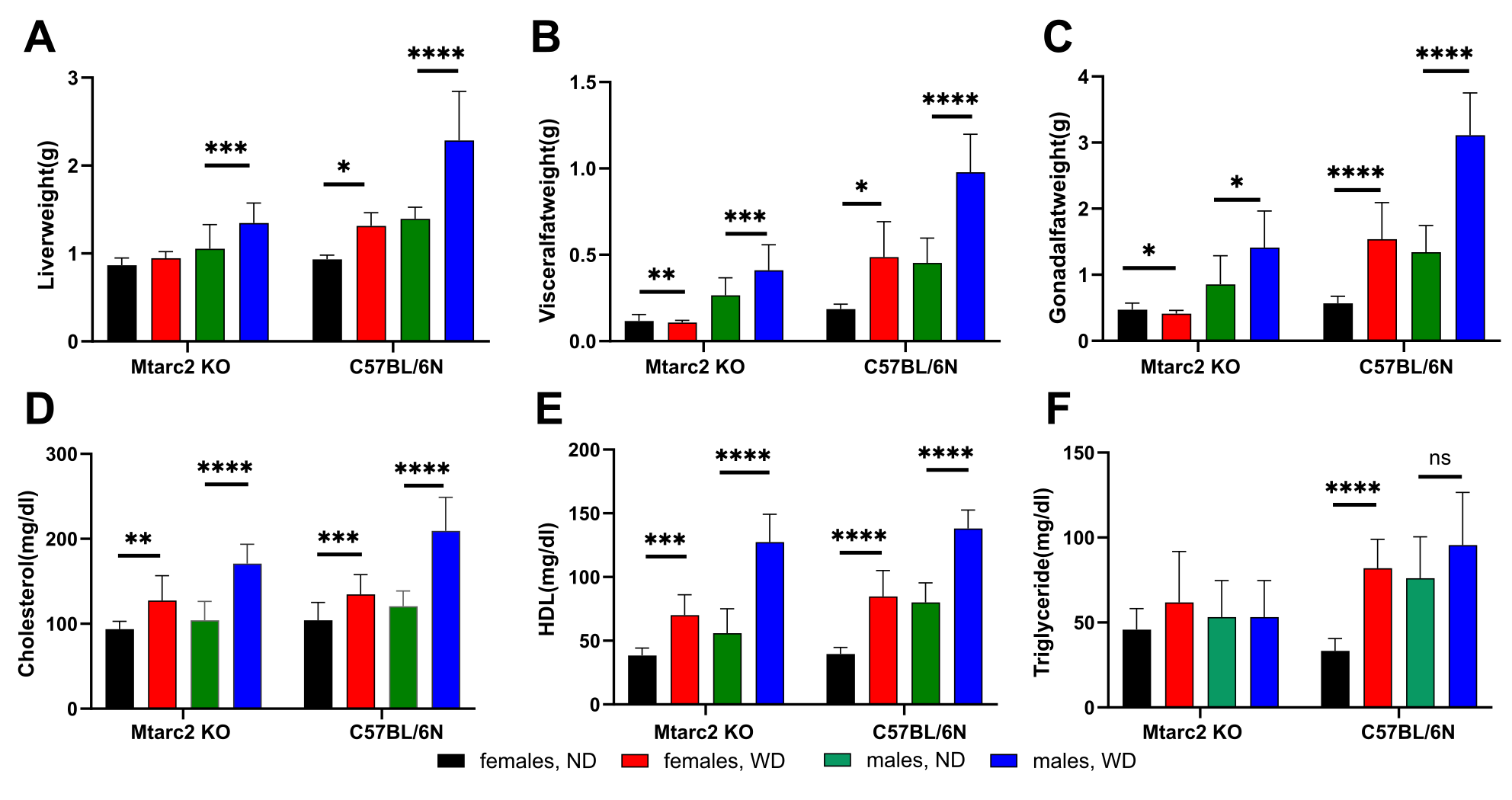

Supplement: Supplementary file 3 — Supplementary Material 3: Fig. 3. Comparisons of liver weights (A), visceral fat weights (B), gonadal fat weights (C), serum total cholesterol concentrations (D), low-density lipoprotein cholesterol (HDL) concentrations (E) and triglyceride concentrations (F) between groups of Mtarc2-KO and C57BL/6N mice that were fed a normal diet (ND) and those fed a Western Diet (WD) for 16 weeks. Values are expressed as mean ± SD, n = 8–10 mice per group. * p < 0.05, ** p < 0.01, *** p < 0.001 and **** p < 0.0001. [file 12263_2025_772_MOESM3_ESM.tiff]

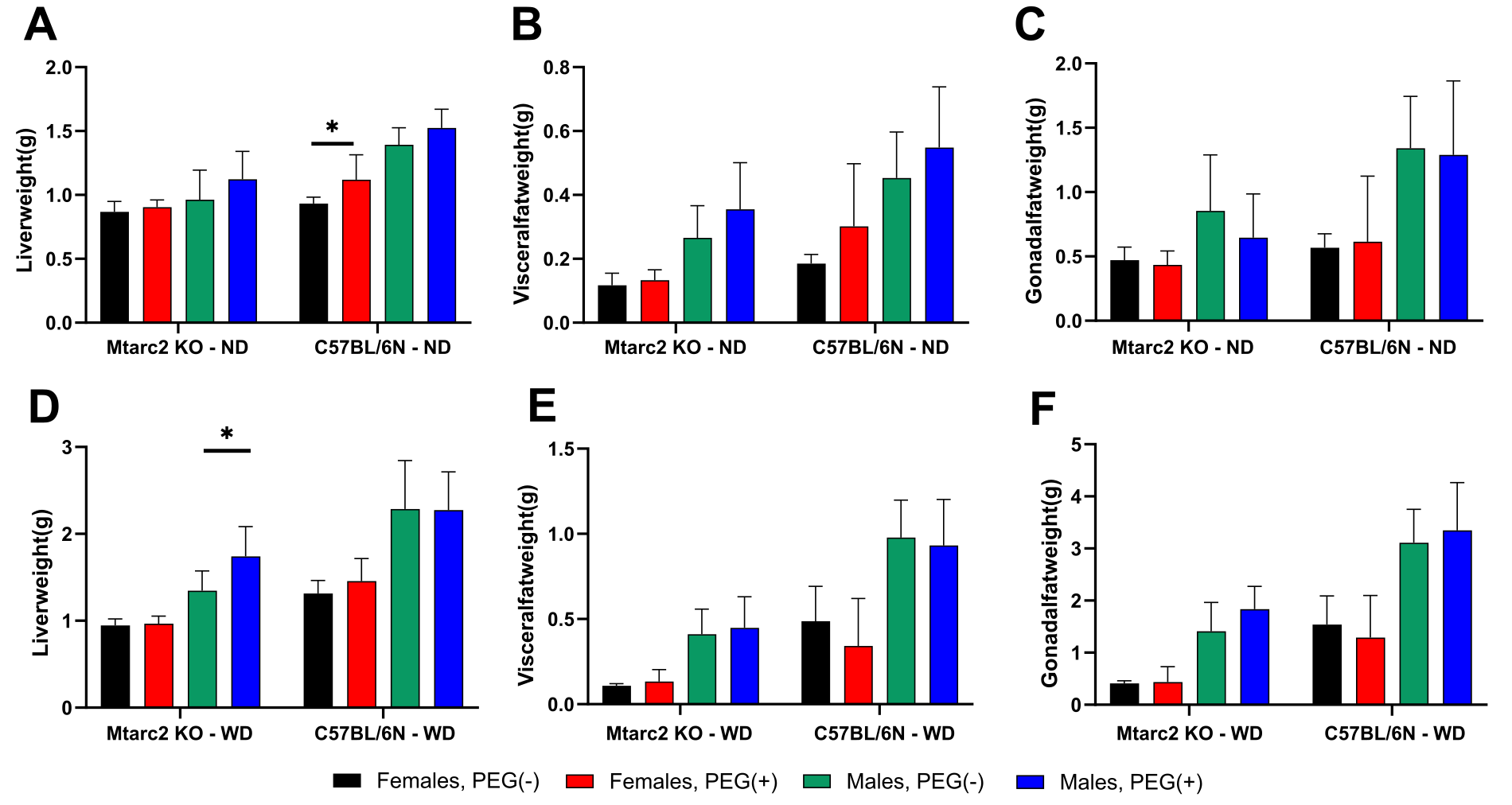

Supplement: Supplementary file 4 — Supplementary Material 4: Fig. 4. Comparisons of liver weights (A), visceral fat weights (B), and gonadal fat weights (C) between groups of mice that were not treated [PEG (-)] and were treated with PEG [PEG (+)] at 4 monthly intervals. Values are expressed as mean ± SD, n = 8–10 mice per group. * p < 0.05. [file 12263_2025_772_MOESM4_ESM.tiff]

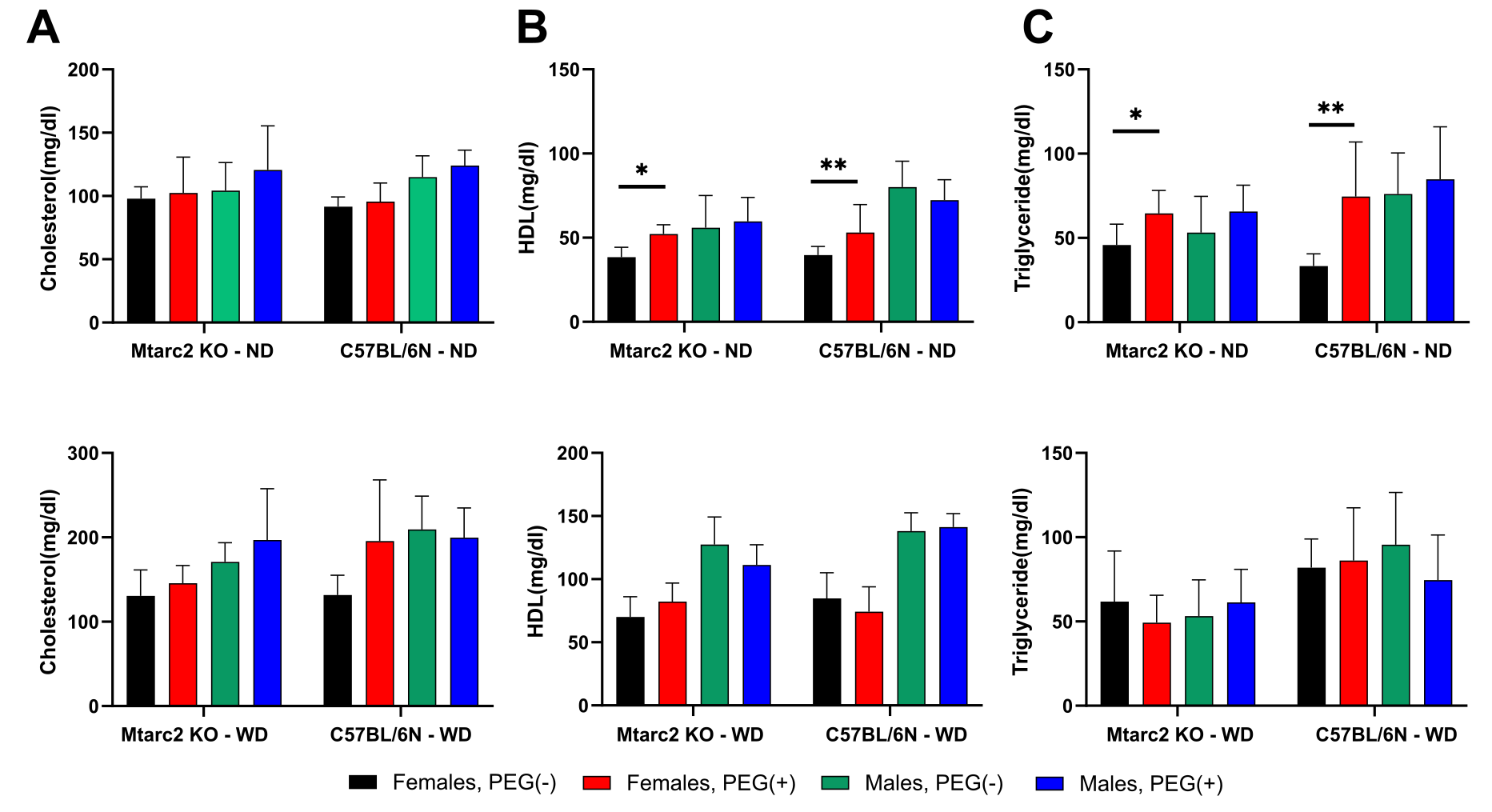

Supplement: Supplementary file 5 — Supplementary Material 5: Fig. 5. Comparisons of serum concentrations of total cholesterol (A), low-density lipoprotein cholesterol (HDL) (B), and triglyceride (C) between mice groups that were not treated [PEG (-)] and were treated with PEG [PEG (+)] at 4 monthly intervals. Values are expressed as mean ± SD, n = 8–10 mice per group. * p < 0.05, ** p < 0.01. [file 12263_2025_772_MOESM5_ESM.tiff]

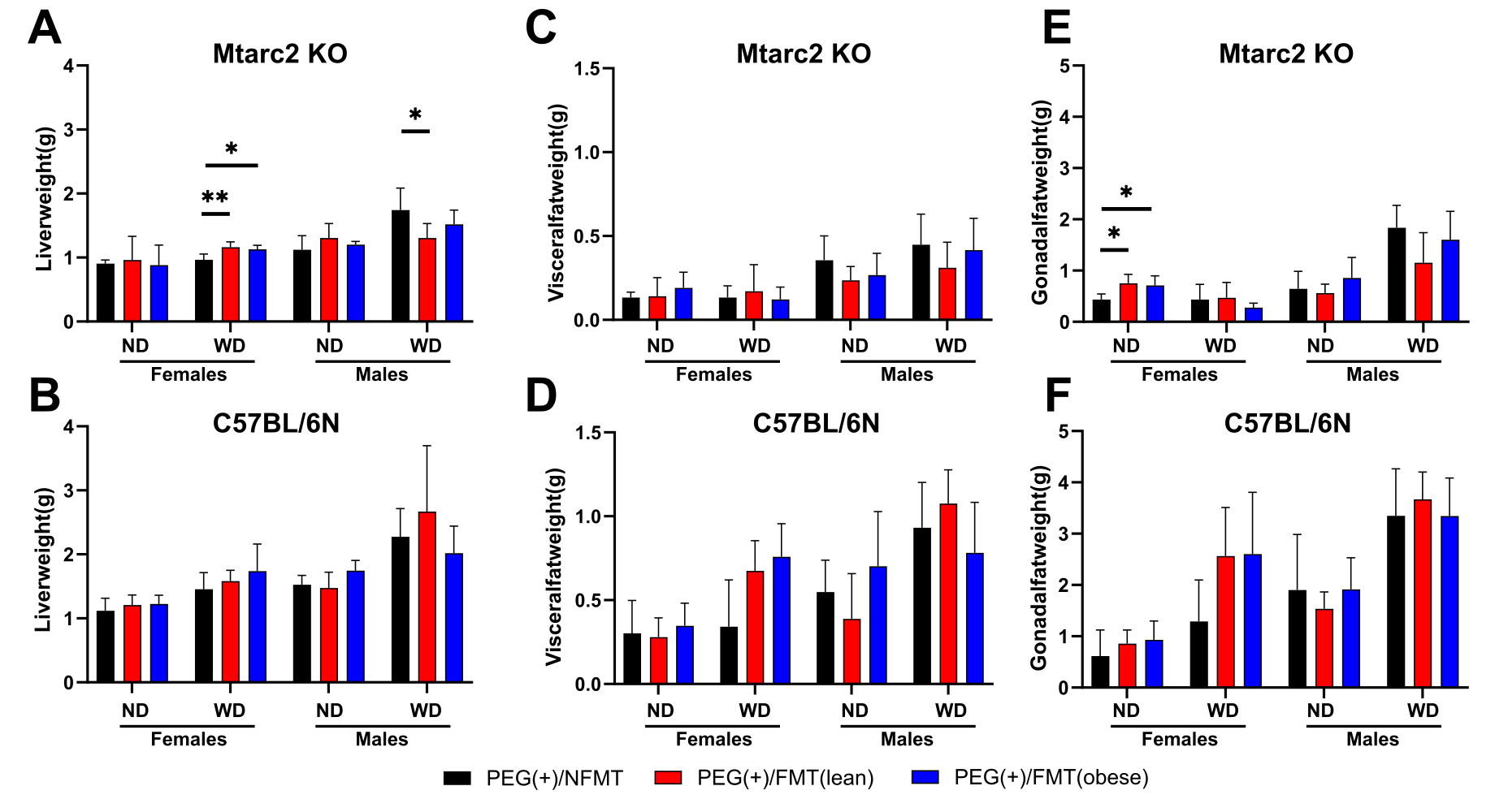

Supplement: Supplementary file 6 — Supplementary Material 6: Fig. 6. Comparisons of liver, visceral fat and gonadal fat weights of Mtarc2-KO (A,C,E) and C57BL/6N (B,D,F) mice fed ND or WD that were only PEG treated [PEG/NFMT] and those transplanted with fecal microbiota isolated from lean [PEG/FMT(lean)] of obese [PEG/FMT(obese)] human donors. Values are expressed as mean ± SD, n = 8–10 mice per group. * p < 0.05, ** p < 0.01. [file 12263_2025_772_MOESM6_ESM.tiff]

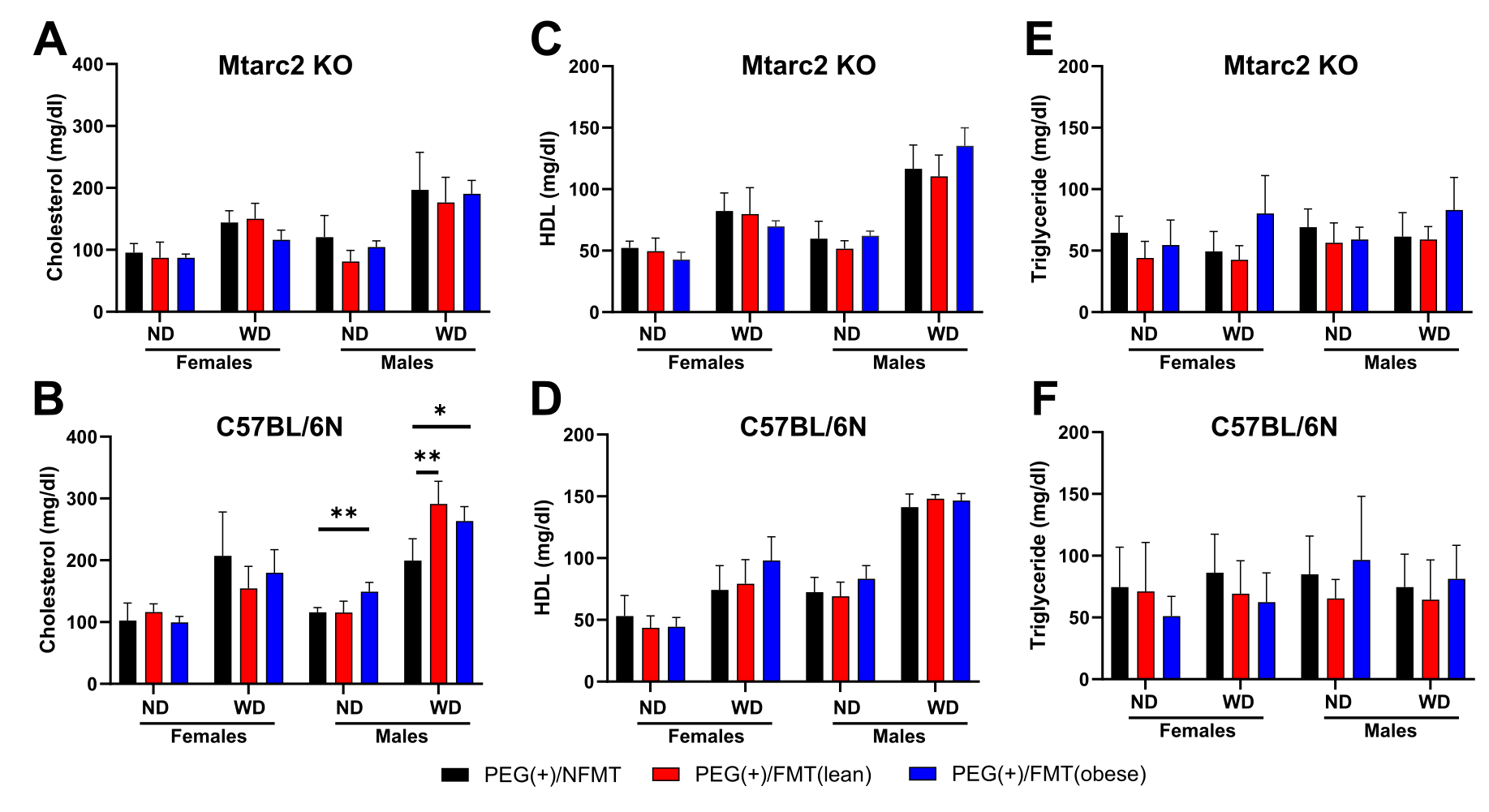

Supplement: Supplementary file 7 — Supplementary Material 7: Fig. 7. Comparisons of serum concentration of total cholesterol, HDL and triglycerides in Mtarc2-KO (A,C,E) and C57BL/6N (B,D,F) mice fed ND or WD that were only PEG treated [PEG/FT(-)] and those transplanted with fecal microbiota isolated from lean (PEG/LFT) of obese (PEG/OFT) human donors. Values are expressed as mean ± SD, n = 8–10 mice per group. * p < 0.05, ** p < 0.01. [file 12263_2025_772_MOESM7_ESM.tiff]

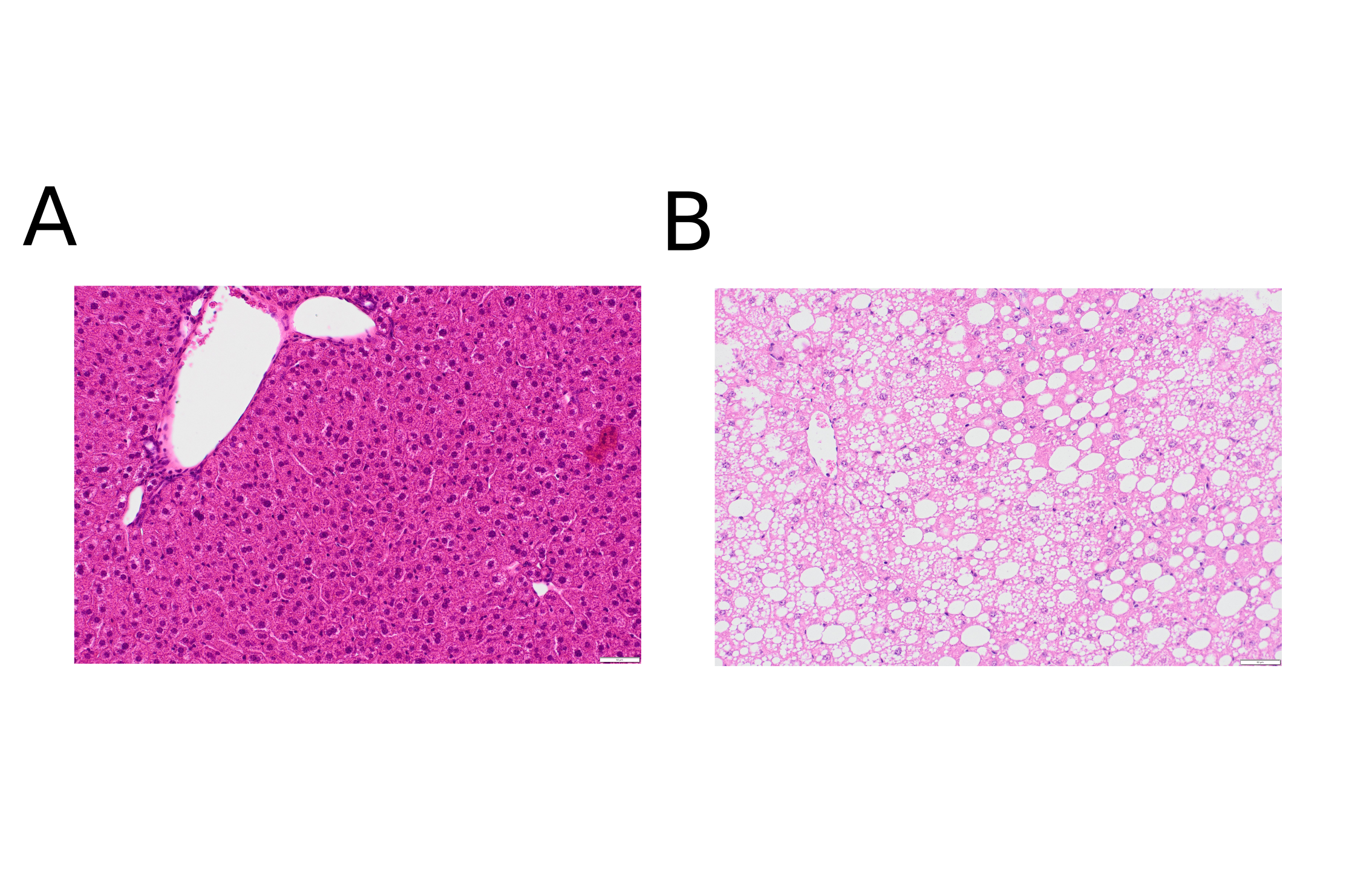

Supplement: Supplementary file 8 — Supplementary Material 8: Fig. 8. Histopathology examination showing normal (A) and fatty liver (B). [file 12263_2025_772_MOESM8_ESM.tiff]

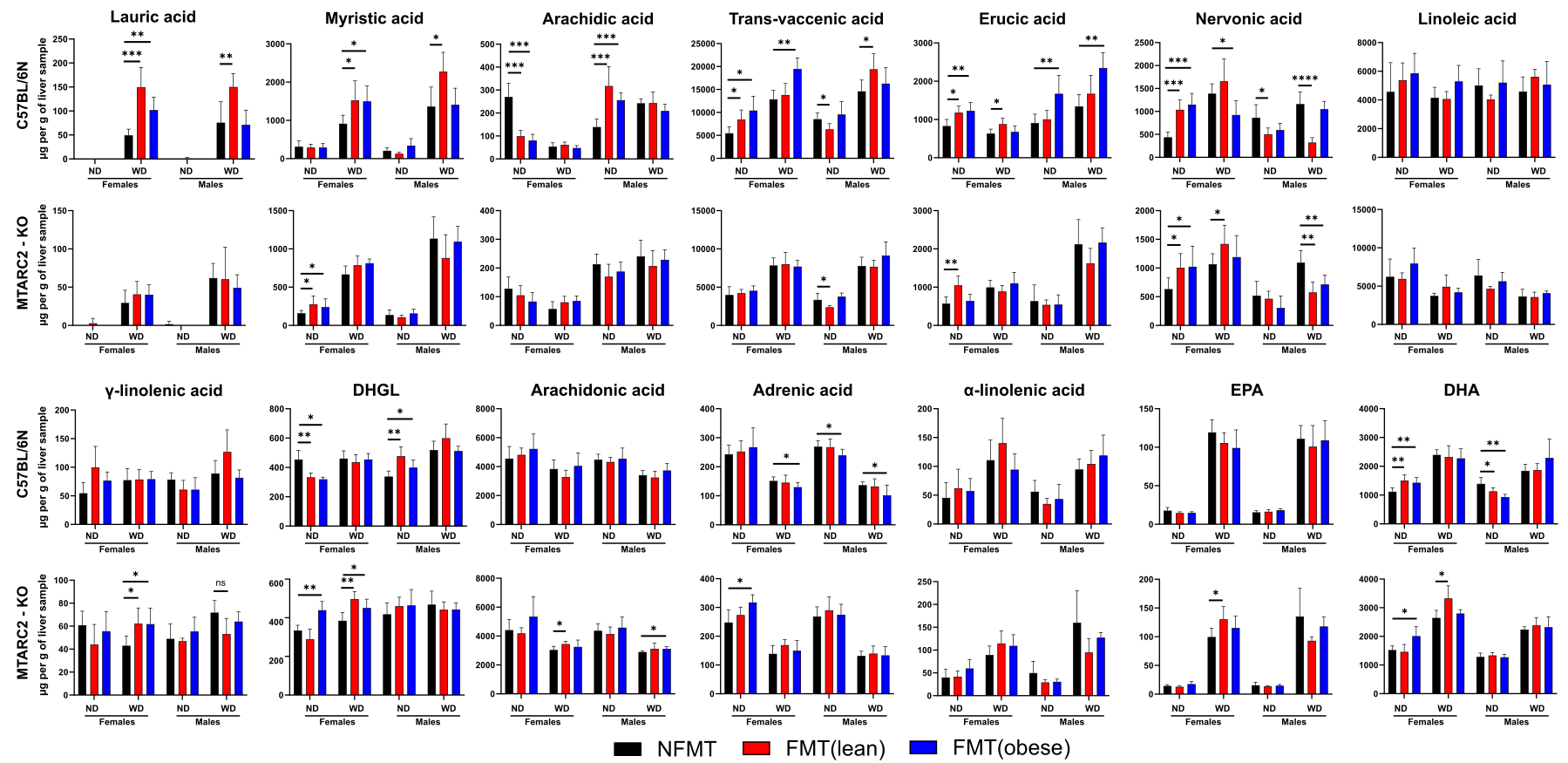

Supplement: Supplementary file 9 — Supplementary Material 9: Fig. 9. Comparisons of liver fatty acid concentrations in Mtarc2-KO and C57BL/6N mice fed ND or WD that were only PEG treated [PEG/NFMT] and those transplanted with fecal microbiota isolated from lean [PEG/FMT(lean)] of obese [PEG/FMT(obese)] human donors. Values are expressed as mean ± SD, n = 8–10 mice per group. * p < 0.05, ** p < 0.01. [file 12263_2025_772_MOESM9_ESM.tiff]

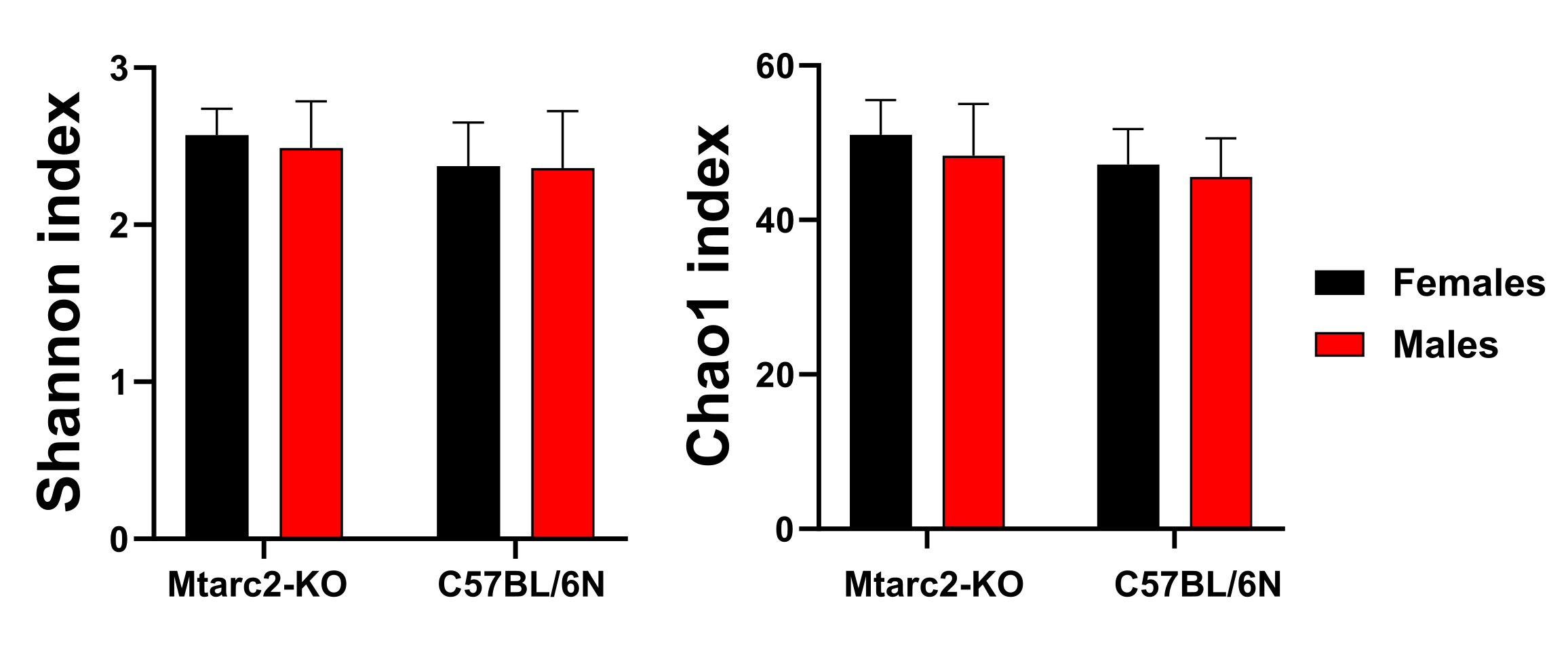

Supplement: Supplementary file 10 — Supplementary Material 10: Fig. 10. ∝-diversity analyzed by the Shannon and Chao1 indexes in fecal samples collected at the beginning (T0) of experiment. [file 12263_2025_772_MOESM10_ESM.tiff]

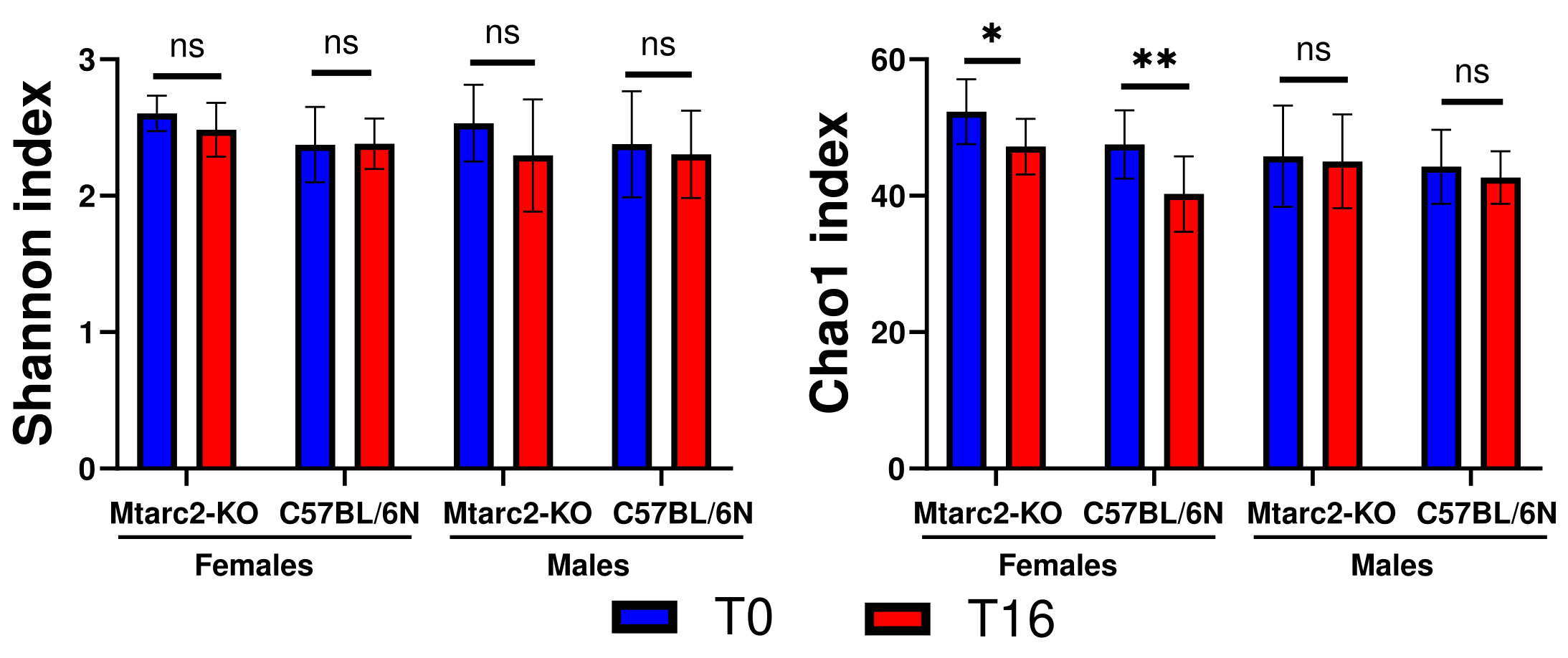

Supplement: Supplementary file 11 — Supplementary Material 11: Fig. 11. ∝-diversity analyzed by the Shannon and Chao1 indexes in fecal samples collected at the beginning (T0) and the end (T16) of experiments from Mtarc2-KO and C57BL/6N mice fed WD mice fed a WD. Statistical significance: * p < 0.05; ** p < 0.01. [file 12263_2025_772_MOESM11_ESM.tiff]

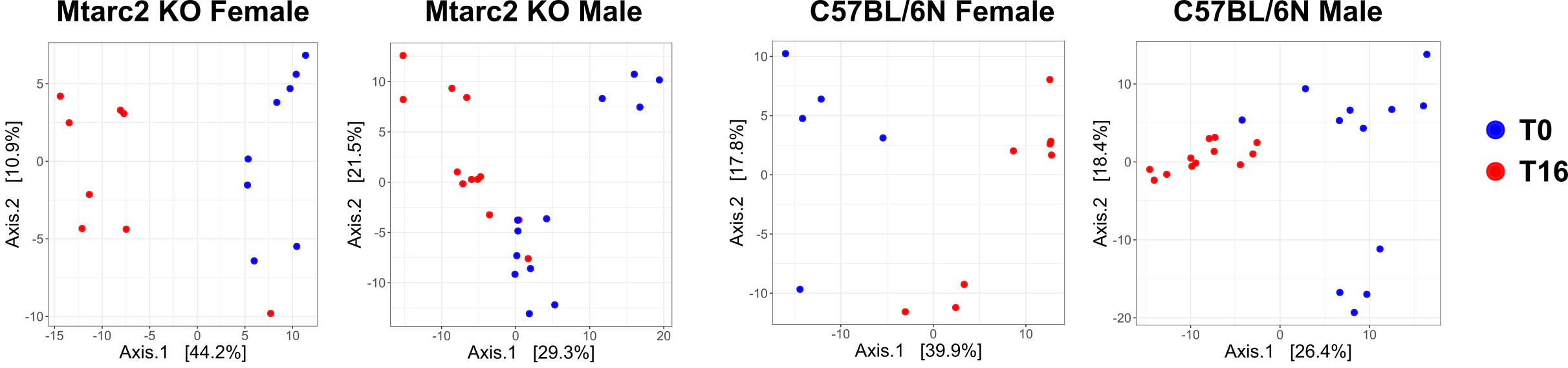

Supplement: Supplementary file 12 — Supplementary Material 12: Fig. 12. Principal coordinate analysis (PCoA) using the Euclidean metric of fecal samples collected at the beginning (T0) and the end (T16) of experiments from Western Diet (WD)-fed mice. Each dot represents a single sample. [file 12263_2025_772_MOESM12_ESM.tiff]

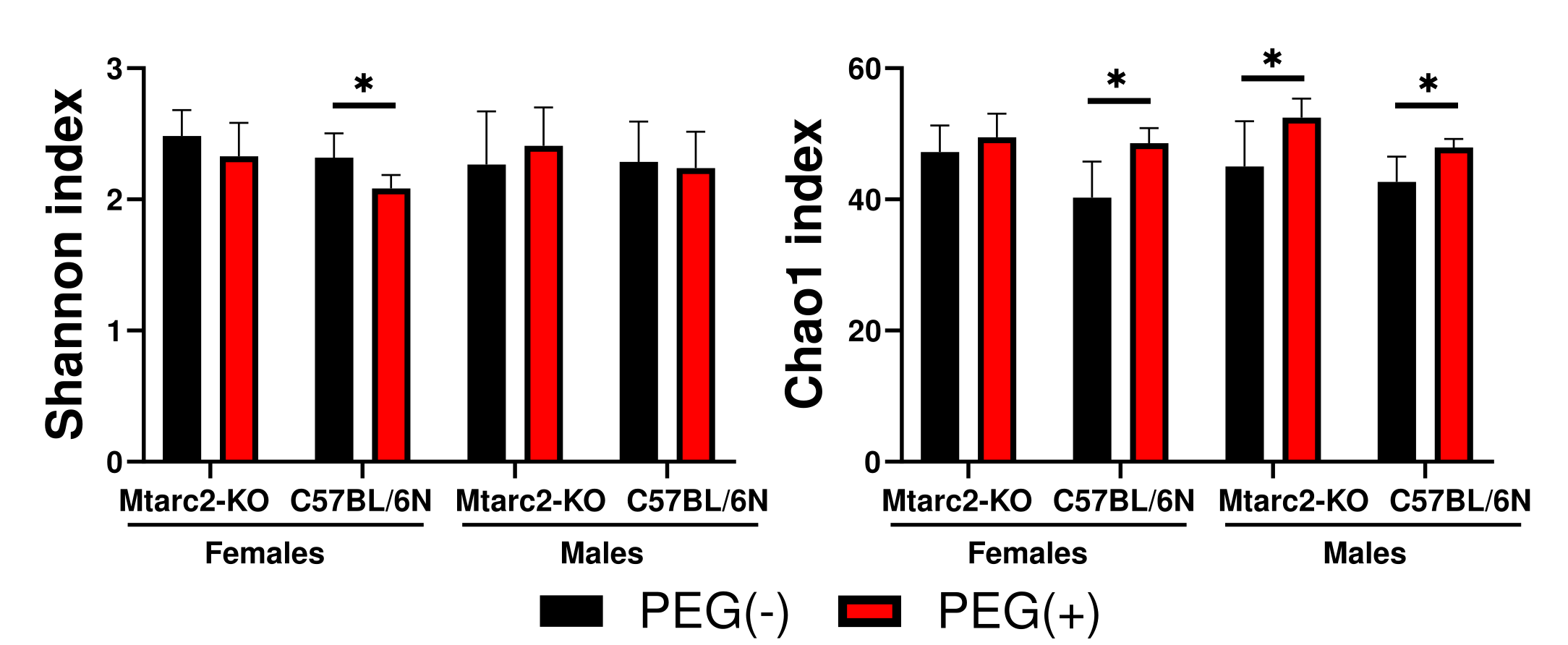

Supplement: Supplementary file 13 — Supplementary Material 13: Fig. 13. ∝-diversity analyzed by the Shannon and Chao1 indexes in fecal samples collected at T16 from PEG(-) and PEG(+) groups of mice fed a WD. Statistical significance: * p < 0.05; ** p < 0.01. [file 12263_2025_772_MOESM13_ESM.tiff]

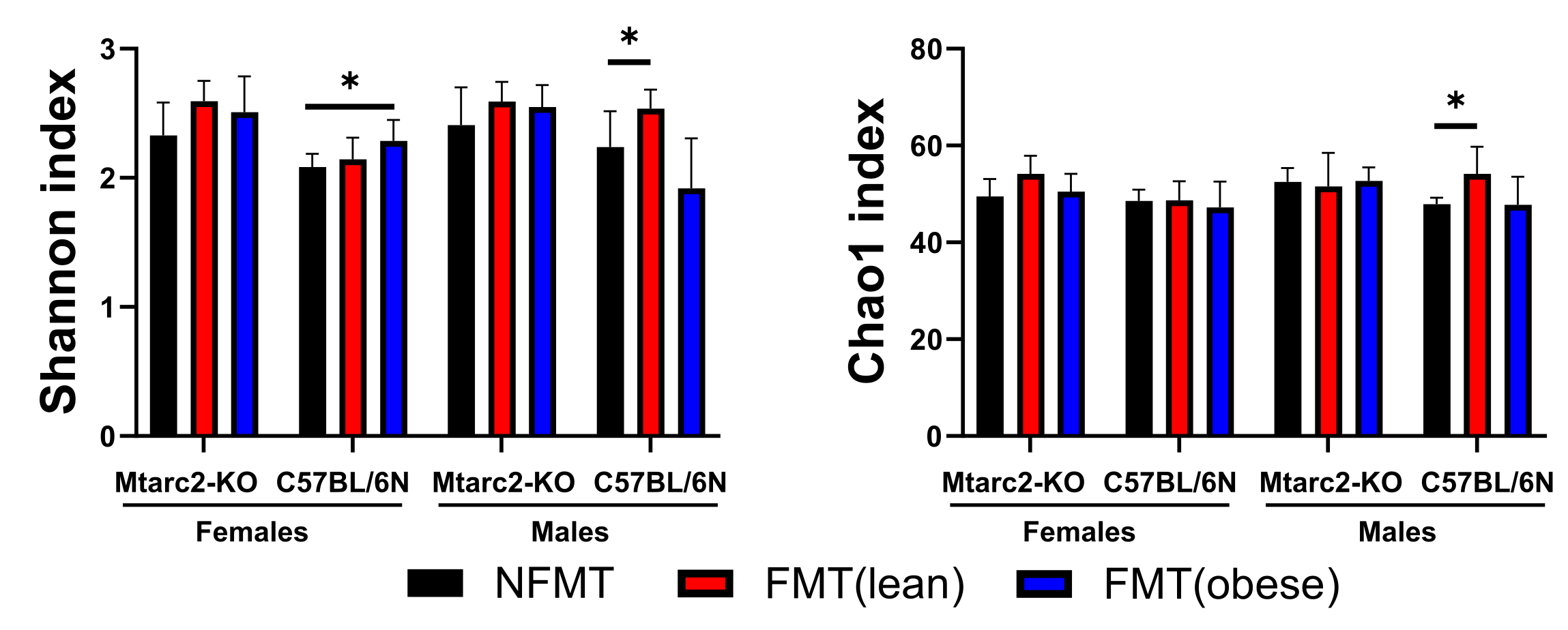

Supplement: Supplementary file 14 — Supplementary Material 14: Fig. 14. ∝-diversity analyzed by the Shannon and Chao1 indexes in fecal samples collected at T16 from WD-fed mice not transplanted (NFMT) and transplanted with fecal extracts of lean [FMT(lean)] and obese [FMT (obese)] human donors. Statistical significance: * p < 0.05. [file 12263_2025_772_MOESM14_ESM.tiff]

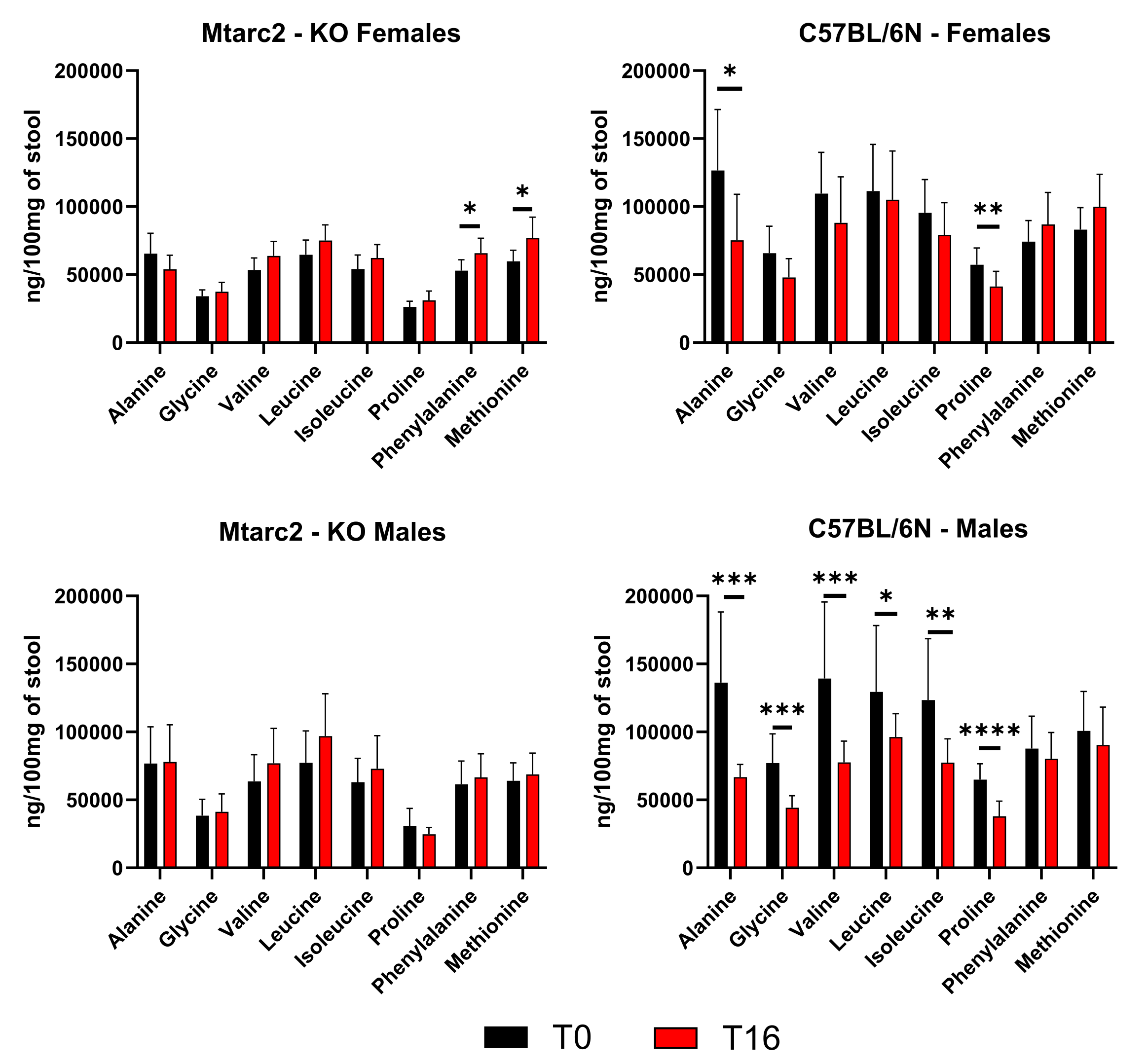

Supplement: Supplementary file 15 — Supplementary Material 15: Fig. 15. Comparison of relative abundance of amino acids between T16 and T0 in mice fed WD. Statistical significance: * p < 0.05; ** p < 0.01; *** p < 0.001; **** p < 0.0001. [file 12263_2025_772_MOESM15_ESM.tiff]

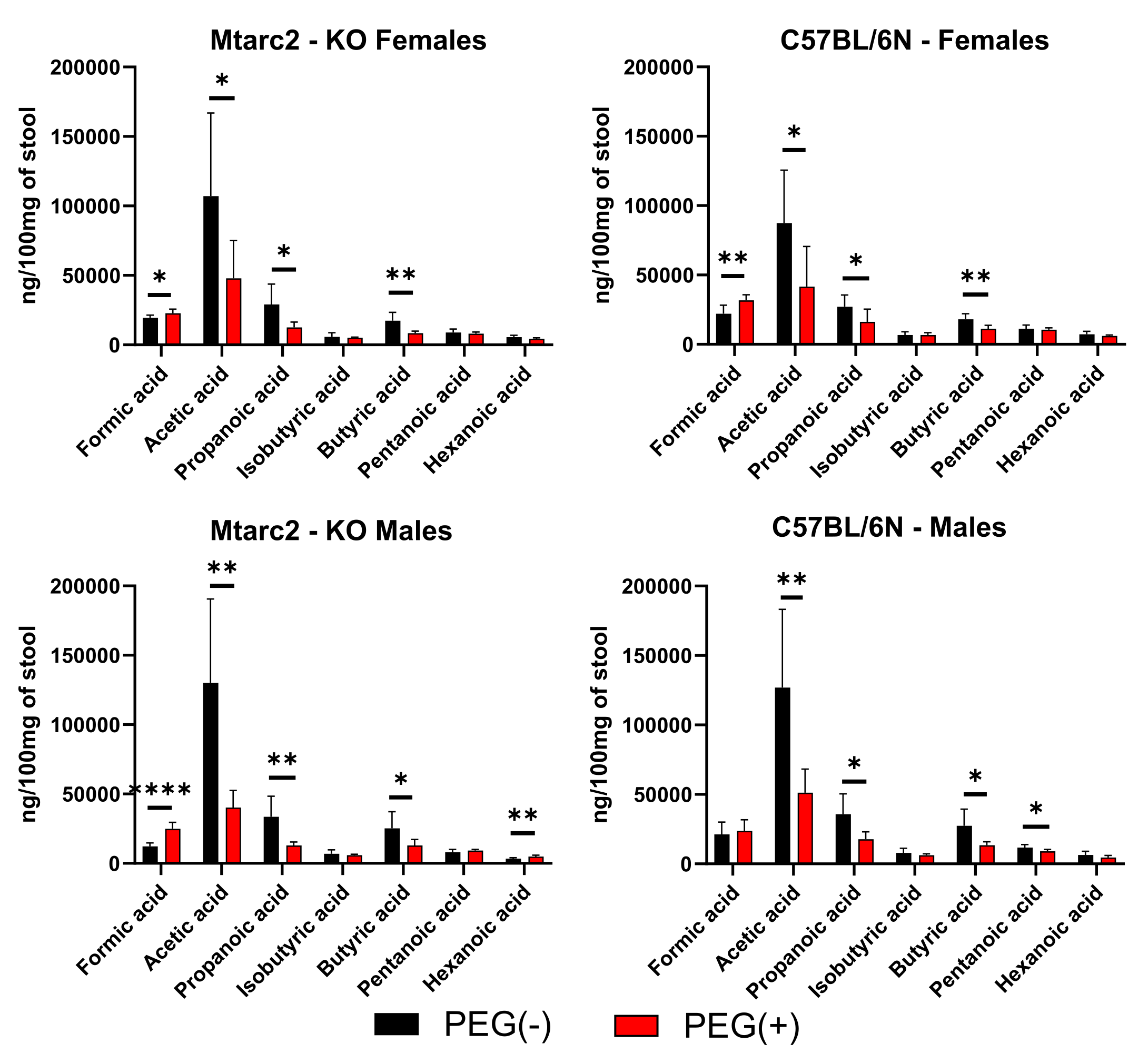

Supplement: Supplementary file 16 — Supplementary Material 16: Fig. 16. Comparison of relative abundance of short chain fatty acids (SCFAs) between PEG(-) and PEG(+) WD fed groups at the end of the experiment. Statistical significance: * p < 0.05; ** p < 0.01; **** p < 0.0001. [file 12263_2025_772_MOESM16_ESM.tiff]

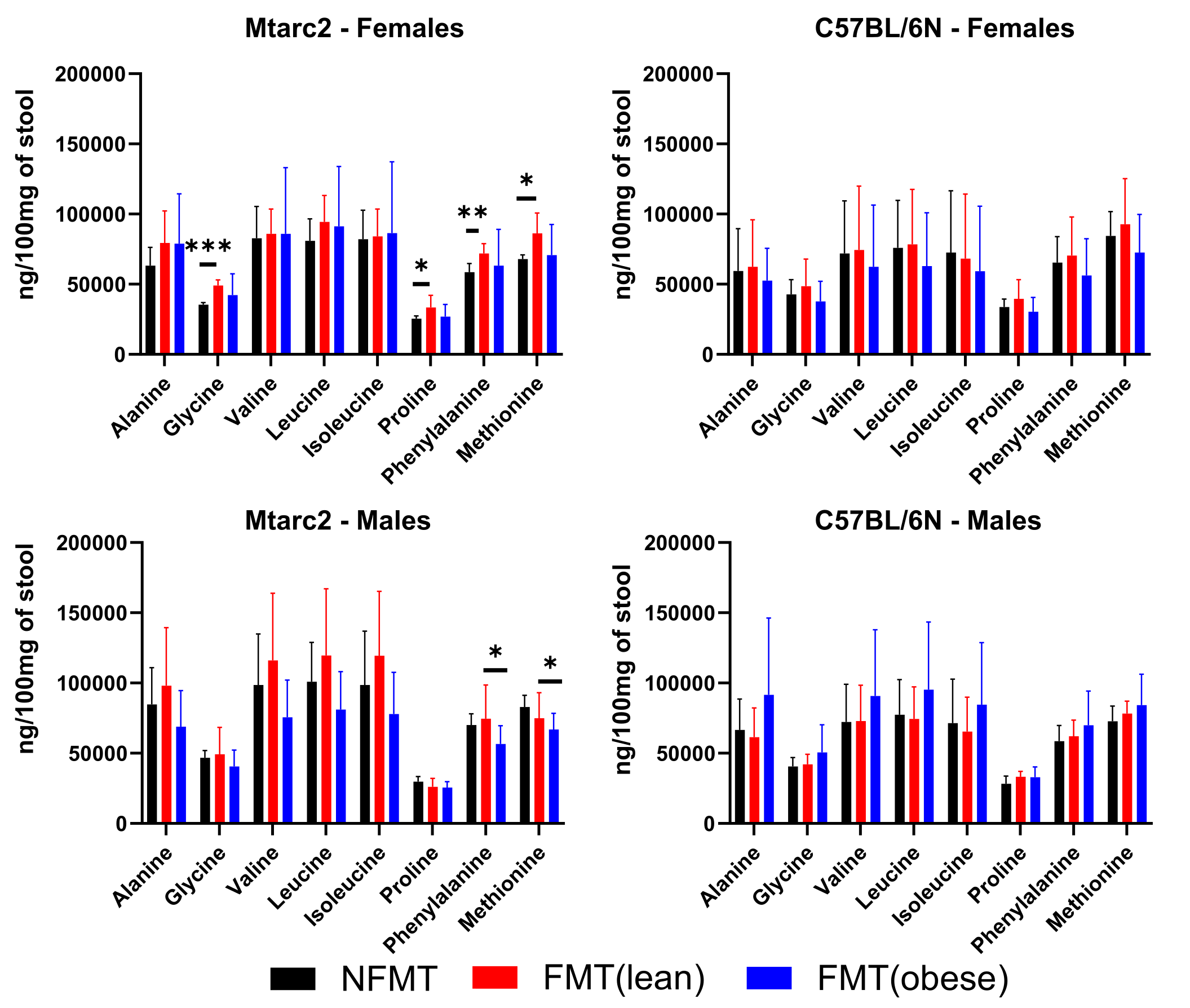

Supplement: Supplementary file 17 — Supplementary Material 17: Fig. 17. Comparison of relative abundance of amino acids (A.As) tested at the end of experiment between WD-fed mice which were not transplanted (NFMT) and those transplanted with fecal extracts of lean [FMT(lean)] and obese [FMT(obese)] human donors. Statistical significance: * p < 0.05; ** p < 0.01; *** p < 0.001. [file 12263_2025_772_MOESM17_ESM.tiff]

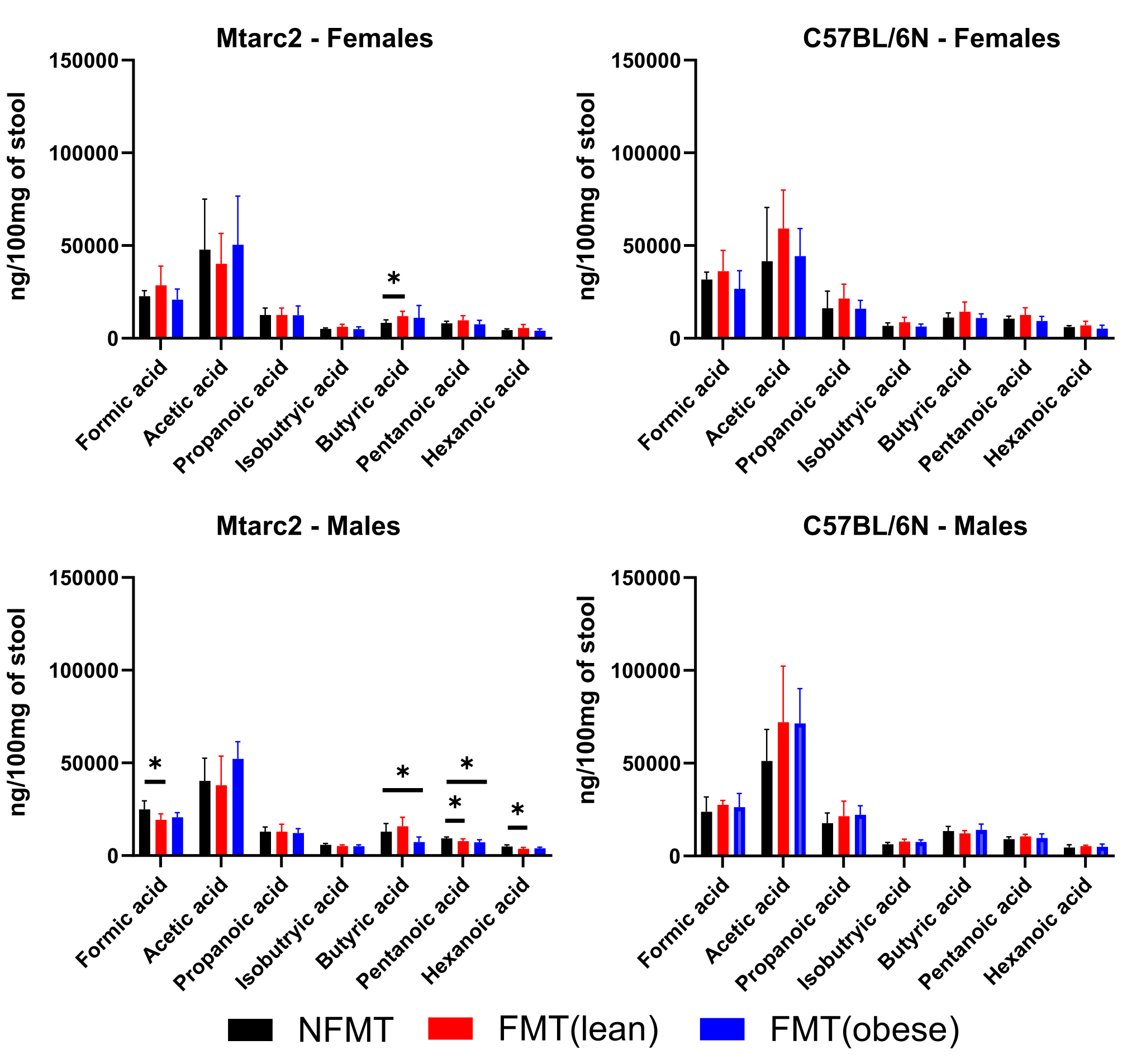

Supplement: Supplementary file 18 — Supplementary Material 18: Fig. 18. Comparison of relative abundance of short chain fatty acids (SCFAs) tested at the end of experiment between WD-fed mice which were not transplanted (NFMT) and those transplanted with fecal extracts of lean [FMT(lean)] and obese [FMT(obese)] human donors. Statistical significance: * p < 0.05. [file 12263_2025_772_MOESM18_ESM.tiff]
