# Supplementary material for: Western Diet and fecal microbiota transplantation alter phenotypic, liver fatty acids, and gut metagenomics and metabolomics in Mtarc2 knockout mice
Source: Genes Nutr. 2025 May 29;20:13. doi: 10.1186/s12263-025-00772-x (PMC12121045; doi:10.1186/s12263-025-00772-x)
Supplement: Supplementary file 19 — Supplementary Material 19: Table 1. Effects of high fat diet (WD) feeding for 16 weeks on individual hepatic fatty acid concentrations (µg/g of sample) in Mtarc2-KO and C57BL/6N females and males. [file 12263_2025_772_MOESM19_ESM.docx]

Additional Table 1. Effects of high fat diet (WD) feeding for 16 weeks on individual hepatic fatty acid concentrations (μg/g of sample) in Mtarc2-KO and C57BL/6N females and males.

| Fatty acid | Mtarc2-KO | | C57BL/6N | |
| --- | --- | --- | --- | --- |
|  | Females | Males | Females | Females |
|  | Mean of ND  vs.  Mean of WD | Mean of ND  vs.  Mean of WD | Mean of ND  vs.  Mean of HFD | Mean of ND  vs.  Mean of WD |
| lauric acid | 0 vs. 33,3**** | 3,3 vs. 79,1** | 0 vs. 46,2**** | 0 vs. 134,1** |
| myristic acid | 141,7 vs. 470,2**** | 165,2 vs. 1485,0**** | 161,0 vs. 715,9**** | 166,7 vs. 870,4** |
| arachidic acid | 3,1 vs. 3,2 | 115,6 vs. 190,5 | 72,9 vs. 35,6**** | 159,9 vs. 471,2** |
| trans-vaccenic acid | 1695,0 vs. 3586,0*** | 1763,0 vs. 4286,0*** | 2119,0 vs. 4443,0*** | 1852,0 vs. 6560,0*** |
| erucic acid | 461,1 vs. 654,1** | 714,2 vs. 644,7 | 496,6 vs. 605,5 | 736,1 vs. 918,4 |
| nervonic acid | 5844,0 vs. 4672,0* | 5391,0 vs. 3946,0* | 7562,0 vs. 3801,0*** | 5942,0 vs. 4318,0* |
| linoleic acid | 2,8 vs. 3,9 | 24,7 vs. 106,1*** | 2,9 vs. 3,4 | 41,1 vs. 890,6**** |
| γ-linolenic acid | 114,0 vs. 98,0 | 121,2 vs. 111,5 | 143,3 vs. 100,4** | 84,5 vs. 200,8*** |
| di-homo-gamma-linolenic acid | 4164,0 vs. 3366,0 | 3974,0 vs. 3541,0 | 5469,0 vs. 3080,0** | 4097,0 vs. 5795,0* |
| arachidonic acid | 43,7 vs. 54,3 | 31,5 vs. 48,6 | 64,0 vs. 59,0 | 47,6 vs. 61,6 |
| adrenic acid | 378,1 vs. 238,9**** | 415,2 vs. 212,7**** | 482,8 vs. 218,7**** | 395,4 vs. 249,4** |
| α-linolenic acid | 4,7 vs. 47,8**** | 14,8 vs. 65,3*** | 17,5 vs. 57,8**** | 7,0 vs. 117,2**** |
| eicosapentaenoic acid | 10,4 vs. 207,5**** | 4,6 vs. 179,8**** | 4,6 vs. 173,0**** | 18,2 vs. 151,4**** |
| docosahexaenoic acid | 2691,0 vs. 5474,0**** | 2629,0 vs. 4359,0**** | 3114,0 vs. 4661,0**** | 2095,0 vs. 4546,0**** |

* p < 0.05, ** p < 0.01, *** p <0.001 and **** p < 0.0001.
