# Supplementary material for: Western Diet and fecal microbiota transplantation alter phenotypic, liver fatty acids, and gut metagenomics and metabolomics in Mtarc2 knockout mice
Source: Genes Nutr. 2025 May 29;20:13. doi: 10.1186/s12263-025-00772-x (PMC12121045; doi:10.1186/s12263-025-00772-x)
Supplement: Supplementary file 20 — Supplementary Material 20: Table 2. Effects of intestinal cleansing by PEG treatment on individual liver fatty acid concentrations (µg/gm of sample) in mARC2-KO and C57BL6/W females and males. [file 12263_2025_772_MOESM20_ESM.doc]

Additional Table 2. Effects of intestinal cleansing by PEG treatment on individual liver fatty acid concentrations (μg/gm of sample) in mARC2-KO and C57BL6/W females and males.

| Fatty acid | mARC2-KO mice | | | | B6 mice | | | |
| --- | --- | --- | --- | --- | --- | --- | --- | --- |
|  | Females, ND | Females, HFD | Males, ND | Males, HFD | Females, ND | Females, HFD | Males, ND | Males, HFD |
|  | PEG (-) vs. PEG (+) | PEG (-) vs. PEG (+) | PEG (-) vs. PEG (+) | PEG (-) vs. PEG (+) | PEG (-) vs. PEG (+) | PEG (-) vs. PEG (+) | PEG (-) vs. PEG (+) | PEG (-) vs. PEG (+) |
| lauric acid | 0 vs. 0 | 33,29 vs. 29,31 | 3,29 vs. 1,62 | 79,13 vs. 61,94 | 0 vs. 0 | 46,18 vs. 49,31 | 0 vs. 0 | 134,1 vs. 75,74 |
| myristic acid | 141,7 vs. 159,4 | 470,2 vs. 667,3** | 165,2 vs. 136,2 | 1485 vs. 1134 | 161 vs. 311,7* | 715,9 vs. 914,8 | 166,7 vs. 206,1 | 870,4 vs. 1361 |
| arachidic acid | 3,097 vs. 128**** | 3,229 vs. 57,27*** | 115,6 vs. 213,6*** | 190,5 vs. 240,7 | 72,87 vs. 270,3**** | 35,63 vs. 53,33 | 159,9 vs. 138,8 | 471,2 vs. 242,1* |
| trans-vaccenic acid | 1695 vs. 3974*** | 3586 vs. 7836**** | 1763 vs. 3339*** | 4286 vs. 7763*** | 2119 vs. 5472**** | 4443 vs. 12864**** | 1852 vs. 8531**** | 6560 vs. 14591**** |
| erucic acid | 2,843 vs. 570,4**** | 3,87 vs. 992,6**** | 24,7 vs. 634,4**** | 106,1 vs. 2119**** | 2,938 vs. 829,9**** | 3,375 vs. 631,3**** | 41,05 vs. 907,1**** | 890,6 vs. 1344* |
| nervonic acid | 114 vs. 632,1**** | 98,02 vs. 1066*** | 121,2 vs. 519,3*** | 111,5 vs. 1092**** | 143,3 vs. 437,4**** | 100,4 vs. 1386**** | 84,49 vs. 864**** | 200,8 vs. 1159**** |
| linoleic acid | 4164 vs. 6215 | 3366 vs. 3718 | 3974 vs. 6388** | 3541 vs. 3659 | 5469 vs. 4560 | 3080 vs. 4143* | 4097 vs. 5013 | 5795 vs. 4589 |
| γ-linolenic acid | 43,74 vs. 60,89* | 54,33 vs. 42,96 | 31,48 vs. 48,99* | 48,57 vs. 71,83 | 63,99 vs. 54,59 | 59,04 vs. 77,48 | 47,61 vs. 78,43* | 61,63 vs. 89,14 |
| di-homo-gamma-linolenic acid | 461,1 vs. 334,4** | 654,1 vs. 384,5**** | 714,2 vs. 417,8**** | 644,7 vs. 467,9 | 496,6 vs. 454,6 | 605,5 vs. 460,3 | 736,1 vs. 338*** | 918,4 vs. 518,4**** |
| arachidonic acid | 5844 vs. 4412* | 4672 vs. 3039* | 5391 vs. 4366 | 3946 vs. 2894* | 7562 vs. 4560** | 3801 vs. 3838 | 5942 vs. 4478* | 4318 vs. 3411 |
| adrenic acid | 378,1 vs. 247,9*** | 238,9 vs. 138,7**** | 415,2 vs. 268,8**** | 212,7 vs. 131,4*** | 482,8 vs. 243,1**** | 218,7 vs. 151,1*** | 395,4 vs. 269,3** | 249,4 vs. 136,4*** |
| α-linolenic acid | 4,729 vs. 39,84*** | 47,79 vs. 89,33** | 14,79 vs. 49,61** | 65,3 vs. 159,9** | 17,51 vs. 45,5* | 57,75 vs. 110,8** | 7,03 vs. 56,06**** | 117,2 vs. 94,57 |
| eicosapentaenoic acid | 10,43 vs. 14,28 | 207,5 vs. 99,36**** | 4,64 vs. 15,45** | 179,8 vs. 135,2 | 4,624 vs. 17,7*** | 173 vs. 119,3* | 18,22 vs. 15,38 | 151,4 vs. 110,8 |
| docosahexaenoic acid | 2691 vs. 1521*** | 5474 vs. 2646**** | 2629 vs. 1280**** | 4359 vs. 2240**** | 3114 vs. 1118**** | 4661 vs. 2394**** | 2095 vs. 1392** | 4546 vs. 1841**** |
